# Choerosponins A and B, Two New Cytotoxic Bridged-Ring Ketones and the Determination of Their Absolute Configurations

**DOI:** 10.3390/molecules22040531

**Published:** 2017-03-27

**Authors:** Chang-Wei Li, Bing Han, Bing Cai, Cheng-Bin Cui

**Affiliations:** 1 State Key Laboratory of Toxicology and Medical Countermeasures, Beijing Institute of Pharmacology and Toxicology, Beijing 100850, China; cuicb@126.com; 2 Beijing Institute for Biomedicinal Research, Beijing 100091, China; h3b3@sohu.com (B.H.); caibing05@126.com (B.C.)

**Keywords:** *Choerospondias axillaries*, bridged ring ketones, absolute configuration, cytotoxicity

## Abstract

Bioactivity-directed fractionation of antitumor compounds from the stem barks of *Choerospondias axillaries* (Roxb.) Burtt et Hill (Anacardiaceae) afforded two new cytotoxic bridged-ring ketones, choerosponins A (**1**) and B (**2**), and their structures were elucidated by spectroscopic methods; their stereochemistry was determined by NOE difference experiments, CD spectra and the modified Mosher’s method. Compound **1** has a rare dioxatricyclo skeleton. Flow cytometry and SRB methods were employed to evaluate the antitumor activity of the two compounds against tsFT210, HCT-15, HeLa, A2780 and MCF-7 cell lines, and both of them showed strong cytotoxicity. MTT and paper disc methods were also used to evaluate their anti-hypoxia and antibacterial activities, and both of them showed no apparent activities.

## 1. Introduction

**Figure 1:**
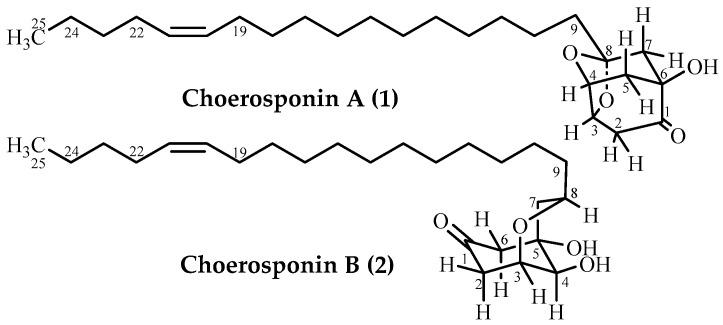
Structures of compounds **1** and **2**.

*Choerospondias axillaries* (Roxb.) Burtt et Hill (Anacardiaceae), the one and only plant species in the genus *Choerospondias*, has been used as a traditional Chinese medicine (TCM) to treat cardiovascular diseases for a long time [1]. So far, phenolic compounds, flavonoids, sterols, organic acids and polysaccharides have been isolated from *C. axillaries*, which displayed diverse biological activities including anti-hypoxia, antibacterial and antitumor activities, and the crude extract of the title plant also showed diverse biological activities, such as anti-arrhythmia, free radical scavenging and immune enhancement activities, etc. [2]. In the course of our screening for antitumor plant materials from the TCMs, we initially found that the EtOAc extract from the stem barks of *C. axillaries* showed strong inhibitory effects on several mammalian cell lines. Previously, we reported some aromatic and flavonoidal constituents from the ethanol extract of its stem bark in our work [3,4,5,6]. Further activity-guided isolation of antitumor compounds from the CHCl_3_ extract partitioned from the 95% ethanol extract led to isolation of two new cytotoxic alkenyled bridged-ring ketones, namely choerosponins A (**1**) and B (**2**) (Figure 1).

## 2. Results

### 2.1. Structure Elucidation of ***1*** and ***2***

Compound **1** was obtained as a white crystalline powder (from CHCl_3_), and its molecular formula C_25_H_42_O_4_ was determined on the basis of its positive HRTOFMS (measured 407.3174 [M + H]^+^, calculated 407.3161 [M + H]^+^) and NMR data (Table 1).

**Figure 2:**
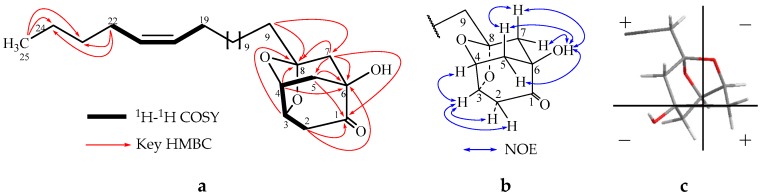
(**a**) ^1^H-^1^H COSY and HMBC correlations of **1**; (**b**) Key NOE correlation of **1**; (**c**) The octant projection of **1**.

The NMR signals of **1** indicated the presence of a carbonyl (C-1), a quaternary oxygenated carbon (C-6), a ketal carbon (C-8), two tertiary oxygenated carbons (CH-3 and CH-4) and four methylene groups (CH_2_-2, CH_2_-5, CH_2_-7 and CH_2_-9). The ^1^H-^1^H COSY correlations of H-2/H-3, H-3/H-4, H-4/H-5, and the HMBC correlations of H_2_-2/C-1, H_2_-5/C-1, H_2_-2/C-6, H-4/C-6 and H_2_-5/C-6 established a partial structure of 3,4,6-oxygenated cyclohexanone (ring C_1_–C_6_) (Figure 2a); HMBC correlations of H_2_-7/C-6, H_2_-7/C-1 and H_2_-7/C-5 showed that a CH_2_-7 was directly located to C-6; HMBC correlations of H_2_-7/C-8, H_2_-9/C-7 and H_2_-9/C-8 indicated that CH_2_-9 was connected to CH_2_-7 through the quaternary ketal carbon C-8; and further HMBC correlations of H-3/C-8 and H-4/C-8 indicated that C-8 was connected to CH-3 and CH-4 through oxygen atoms. Thus, the partial structure of **1** was extended to a rigid dioxatricyclodecanone skeleton (Figure 2a). The ^13^C-NMR showed signals at δ_C_ 29.2−29.7 and δ_C_ 129.8, 129.9 (C-20 and C-21, coupled in HMQC with protons at δ_H_ 5.35), characteristic of the presence of a long side chain with a double bond. The ^1^H-^1^H COSY correlations of H_2_-19/H-20, H-21/H_2_-22 designated that carbons C-19 and C-22 were located at C-20 and C-21, two carbons of the double bond, respectively. The location of the double bond was established through the HMBC correlations of H_2_-22/C-23, H_2_-22/C-24, H_3_-25/C-23 and H_3_-25/C-24, indicating the presence of three carbons (C-22, 23, and 24) between the double bond and the terminal methyl at δ_H_ 0.90 (Figure 2a). The geometry of the double bond was determined as Z by comparison of the chemical shift of C-19 and C-22 (δ_C_: 26.9 and 27.2) with the published values [7,8]. The HMBC correlations of H_2_-9/C-8 and H_2_-10/C-8 suggested the 17C (determined from the formula of C_25_H_42_O_4_) alkenyl chain was located at C-8. This completed the planar structure of **1**.

As displayed in Figure 2, compound **1** has a rigid dioxatricyclodecanone skeleton; 3-*O*- and 4-*O*-groups must be *cis*-oriented, but were *trans*-oriented with 6-OH, which was in agreement with the NOE correlations (Figure 2b). To determine the absolute structure, the CD spectrum was performed, and a positive cotton effect was observed at 278 nm. According to the octant rule [9], based on the octant projection of the stereochemical structure of **1** (Figure 2c), the absolute structure of **1** was identified as 3*S*, 4*R*, 6*S*, 8*R*. Thus the absolute configuration was established.

**Figure 3:**
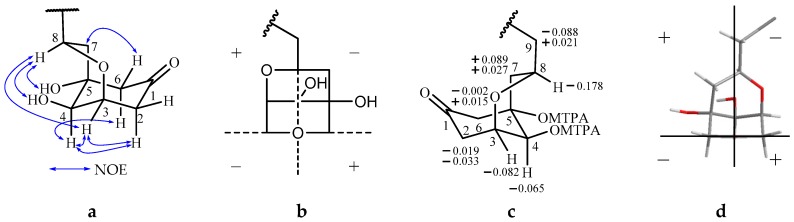
(**a**) The key NOE correlations of **2**; (**b**) The hypothetical octant projection of **2**; (**c**) Values of Δδ (δ_S-MTPA_ - δ_R-MTPA_) of the MTPA esters of **2**; (**d**) The practical octant projection of **2**.

Compound **2** exhibited the molecular formula C_25_H_44_O_4_ established by HRTOFMS (measured 409.3329 [M + H]^+^, calculated 409.3318 [M + H]^+^). Signals observed in the ^1^H and ^13^C-NMR spectra (Table 1) were similar to those observed for **1**, except the signals of C-5, C-6 and C-8. According to the ^1^H-^1^H COSY (H-2/H-3, H-3/H-4) and HMBC correlations (Table 1), a cyclohexanone moiety could be obtained, and C-8 and C-3 were bridged by an oxygen atom, which afforded a oxabicyclonananone skeleton (Figure 3a). The same alkenyl side chain as **1** was also located at C-8. The NOE correlations between H-3 and H-4 suggested that the 3-*O*- group and 4-OH were *cis*-oriented; correlations of H-4/H-6a, H-6e/H-7 indicated that 4-OH and 5-OH were *trans*-oriented; and correlations between H-8 and H-3 indicated that H-8 was at the *pseudo*-axial position of the boat conformation. So the planar structure and relative stereochemistry were fully established.

Like compound **1**, a negative cotton effect was observed in the CD spectrum of **2** at 291.5 nm. In terms of the octant rule [9], the absolute configuration of **2** was initially determined as 3*R*, 4*R*, 5*R* and 8*S*, based on the octant projection **b** in Figure 3. To confirm the absolute configuration of **2**, the modified Mosher’s method was also applied. According to the rule of the modified Mosher’s method [10], the positive Δδ (δ_*S*-MTPA_ - δ_*R*-MTPA_) value of H-7 and the negative Δδ values of H-2 and H-3 indicated that C-4 of **2** has an *S* absolute configuration (Figure 3c). Therefore, the absolute configurations of **2** were further established as 3*S*, 4*S*, 5*S* and 8*R*, which were contradictory to the result determined by the CD spectrum initially. To explore the reason for the contradiction, shorter C-O (C_3_-O and C_8_-O) bonds must be taken into account, which will cause a deflection of the octant projection of **2** in the octant region (Figure 3d). According to the octant projection **d** in Figure 3, the absolute configurations obtained will be coincident with that determined by the modified Mosher’s method. Lastly, the absolute configuration of **2** was exactly established as 3*S*, 4*S*, 5*S* and 8*R*.

### 2.2. Biological Activities of ***1*** and ***2***

#### 2.2.1. Antitumor Activity

Cytotoxic activities and the proliferation-inhibiting effect of compounds **1** and **2** were evaluated by the flow cytometry and SRB methods [11] against the tsFT210, HCT-15, HeLa, A2780 and MCF-7 cell lines. Compounds **1** and **2** showed stronger cytotoxicity and apoptosis-inducing activities at high concentrations, but inhibited tsFT210 cells at the G0/G1 phase at low concentrations (see Figure S24 in Supplementary Data). Compound **1** only showed an inhibition effect against HCT-15 and HeLa cell lines to some extent, but **2** showed a stronger inhibition effect on the above four human cancer cell lines; the IC_50_ values of the proliferation-inhibiting effect are given in Table 2 and Table 3. The positive control, 5-FU, inhibited the above four cancer cells with inhibitory rates between 35%−50% at 100 μg/mL (769 μM).

#### 2.2.2. Anti-Hypoxia Effects

To evaluate the anti-hypoxia activities, ECV304 cells were used as test cells for **1** and **2** by the MTT method [1]. The MTT assays showed that the cell viabilities of the above cell lines treated with **1** and **2** were not increased at 50 µg/mL, which suggested that **1** and **2** exhibited no anti-hypoxia activity (Table 4).

#### 2.2.3. Anti-Bacterial Activity

The antibacterial activities for **1** and **2** were assayed by the 8 mm paper disc method using *Blastomyces albicans* ATCC10231 and *Staphylococcus aureus* ATCC6538. Compounds **1** and **2** did not show any inhibition zones at the concentration of 150 µg/paper disc on the above two bacterial strains.

## 3. Discussion

The ketone functional groups are widely distributed as a structural fragment in many natural products of bridged-ring structures, most of which are chiral compounds [12,13,14]. The absolute configurations of such compounds are often determined by CD spectroscopy in terms of the octant rule for saturated cyclohexanone [9]. CD spectroscopy had been proved to be an indispensable tool for studying the absolute configuration of organic compounds [8], but few papers have reported on the contribution of the oxygen hetero-atom in determining the absolute configuration by CD spectroscopy [15], and this paper afforded another example displaying the effect of the oxygen hetero-atom on determining the absolute configuration of the cyclohexanone based on the octant rule. In addition, a precise absolute configuration will be obtained if some other methods such as the modified Mosher’s method are applied at the same time.

At the beginning of our isolation, the 95% EtOH and CHCl_3_ extracts were evaluated for their cytotoxic activity by the flow cytometry method against tsFT210 cells, and both of them showed strong cytotoxicity and apoptosis-inducing activities. Compounds **1** and **2**, especially **2**, also showed strong cytotoxicity and inhibitory effects against the tested mammalian cancer cell lines. However, the aromatic and flavonoidal constituents isolated in our previous work only showed moderate cytotoxic activity or inhibitory effects on some mammalian cancer cell lines [3,4,5,6]. The results indicated that compounds of **1** and **2** were the main cytotoxic components of the CHCl_3_ extract and the 95% EtOH extract. In addition, though the flavonoidal constituents and galloyl glucose isolated from the EtOAC or BuOH extracts showed anti-hypoxia activity to some extent [3,4,5], compounds **1** and **2** showed no anti-hypoxia activity, which indicated that compounds with phenolic hydroxyl groups would more likely show anti-hypoxia activity.

**Figure 4:**
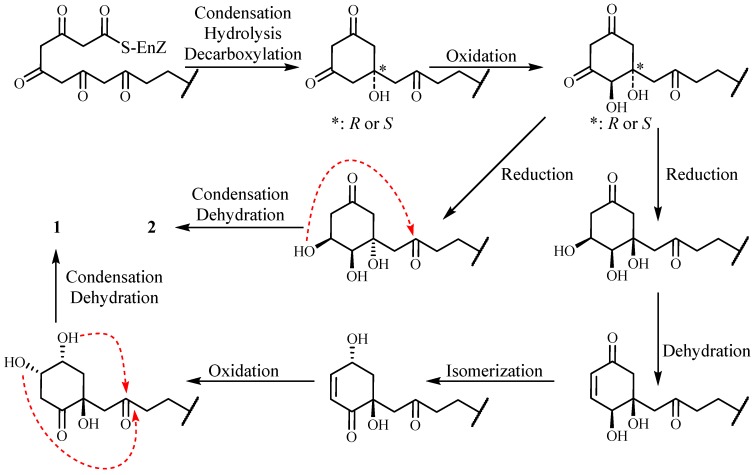
The plausible biosynthetic pathway of compounds **1** and **2**.

From the Anacardiaceae family, cytotoxic n-alkylphenols or derivatives were widely found [7,8,16,17,18]. Compounds such as **1** with a dioxatricyclo skeleton have often been synthesized [19], but the dioxatricyclo skeleton in compound **1** has never been reported. Correia [16] and Walters [20] reported that n-alkylphenols derived from unsaturated fatty acid and unsaturated fatty acid were condensated from acetyl-CoA and malonyl CoA by the acetate-malonate pathway. We presumed that **1** and **2** have a similar biosynthetic pathway (Figure 4).

## 4. Materials and Methods

### 4.1. General Experiment Procedures

Precoated silica gel GF_254_ plates (10 cm × 20 cm, 0.25 mm thickness for analytical TLC and 20 cm × 20 cm, 0.5 mm thickness for preparative TLC; Yantai Chemical Industrial Institute, Yantai, China) were used in TLC and spots were detected under UV light (254 and 365 nm) or by using 10% sulfuric acid reagent. Silica gel H (100–200 mesh, Yantai Chemical Industrial Institute, Yantai, China), YMC GEL^®^ ODS-A-HG (12 nm S-50 μm, YMC Co. Ltd., Kyoto, Japan) and Sephadex™ LH-20 (GE Healthcare, Uppsala, Sweden) were used for column chromatography. The reversed-phase Capcell Pak C18 (UG120Å, 20 mm × 250 mm; Shiseido Co., Ltd., Tokyo, Japan) columns were used in preparative HPLC.

Melting point was measured on a Beijing Tiandiyu X-4 exact micro melting point apparatus (Tiandiyu science and technology Co., Ltd., Beijing, China) and the temperatures were not corrected. Optical rotations were determined on a JASCO P-1020 polarimeter (JASCO electric Co., Ltd., Tokyo, Japan) in MeOH solution and UV spectra were recorded on a Shimadzu UV-2501 PC UV-VIS recording spectrophotometer (Shimadzu corporation, Kyoto, Japan) in MeOH solutions. ESIMS was recorded on an Applied Biosystems API 3000 LC-MS spectrometer (AB SCIEX, Framingham, MA, USA) and HRESIMS was measured on an Agilent 6520 Q-TOF LC-MS spectrometer (Agilent Technologies, Santa Clara, USA). ^1^H and ^13^C-NMR and 2D NMR spectra were taken on a JEOL Eclips-600/400 spectrometer (JASCO electric Co., Ltd., Tokyo, Japan) using TMS as internal standard and chemical shifts are recorded as *δ* values. Bioassay was tested by COULTER EPICS XL flow cytometer and spectra MAX plus plate reader from MD company (Molecular Devices, Silicon Valley, CA, USA).

### 4.2. Plant Material

The stem bark of *C. axillaries* was collected in the Mengla region of Yunnan, China, in september 1999. The plant was identified by Professor Sun Qi-shi and a voucher specimen (No. 050901) was deposited at the Beijing Institute of Pharmacology and Toxicology.

### 4.3. Extraction and Isolation

Stem barks (3.2 kg) was exhaustively extracted with 95% ethanol (25 L, 4 × 7 day), and concentrated in vacuo to give 750 g extract. The extract was suspended in water (4 L), then was partitioned with the same volume of CHCl_3_ (4 L × 4) to give CHCl_3_ extract (60 g), which was submitted to antitumor activity bioassay using flow cytometry method and showed strong cytotoxicity against tsFT210 cell lines. The CHCl_3_ extract (60 g) was subjected to vacuum column chromatography over silica gel (bed: 7.5 × 18.5 cm) eluted with petroleum (P)/acetone (A) to afford six fractions (Fr1−Fr6) based on TLC monitoring and bioassay results. The two main active fractions Fr4 (10.8 g, eluted with P/A 5:1) and Fr5 (13.6 g, eluted with P/A 2:1) were subjected to vocuum column chromatography over silica gel, Sephadex LH-20 gel and PTLC over silica gel, then were further purified using HPLC on a RP-C18 preparative column (eluent, MeOH−H_2_O 85:15) to obtain **1** (*t*_R_ = 45.0 min, 97 mg) and **2** (*t*_R_ = 86.0 min, 106 mg).

### 4.4. Physicochemical Properties and Spectra Data

*Choerosponin A* (**1**): White crystalline powder, m.p. 75−77 °C (MeOH), ${\left[\alpha \right]}_{D}^{31}$ +29.9° (*c* 1.00, CHCl_3_). Positive ion ESI-MS *m*/*z*: 407 [M + H]^+^, 429 [M + Na]^+^, 812 [2M + H]^+^, 835 [2M + Na]^+^. Positive ion HR-ESI-MS *m*/*z*: 407.3174 [M + H]^+^ (calcd. 407.3161). UV λ_max_ (MeOH) nm: end absorption. IR (KBr) *ν*_max_: 3436, 3005, 2925, 2854, 1732, 1465, 1405, 1339, 1113, 1232, 1089, 1045, 998, 827, 648 cm^−1^. CD λ_max_ nm (mdeg) in MeOH at 1.0 mg/mL: 200 (+19.8295), 237 (+0.4396), 278 (+3.1246), 298.5 (−0.0238), 310 (−1.1797), 330 (−0.0099). ^1^H-NMR and ^13^C-NMR data see Table 1.

*Choerosponin B* (**2**): White crystalline powder, m.p. 82−84 °C (MeOH), ${\left[\alpha \right]}_{D}^{31}$ +8.9° (*c* 1.00, CHCl_3_). Positive ion ESI-MS *m*/*z*: 409 [M + H]^+^, 426 [M+NH_4_]^+^, 391 [M + H − H_2_O]^+^. Positive ion HR-ESI-MS *m*/*z*: 409.3329 [M + H]^+^ (calcd. 409.3318). UV λ_max_ (MeOH) nm: end absorption. IR (KBr) *ν*_max_: 3544, 3389, 3026, 2920, 2850, 1715, 1640, 1469, 1460, 1338, 1302, 1232, 1099, 1066, 1042, 1016, 924, 839, 719 cm^−1^. CD λ_max_ nm (mdeg) in MeOH at 1.0 mg/mL: 200 (−17.1618), 238.5 (−0.7975), 291.5 (−3.4174), 339.5 (−0.0790). ^1^H-NMR and ^13^C-NMR data see Table 1.

### 4.5. Preparation of (S)- and (R)-MTPA Esters of ***2***

Two portions (each 2.2 mg, 5.4 μmol) of **2** were treated with (*S*)-(+) and (*R*)-(−)-*α*-methoxy-*α*-trifluoromethylphenylacetyl (MTPA) chloride (9.0 μL, 26.4 μmol) in anhydrous deuterated pyridine (0.5 mL) in separate NMR tubes at room temperature. The reactions were monitored by ^1^H-NMR at certain intervals. The ^1^H-NMR data of the (*S*)- and (*R*)-MTPA esters of **2** were obtained after the reactions were completed three days later, and the assignments of the hydrogen signals were established by ^1^H-^1^H COSY.

*Choerosponin B-(S)-MTPA Ester* (pyridine-*d*_5_, 400 MHz) δ: 3.002 (1H, d, *J* = 14.8 Hz, Ha-2), 2.875 (1H, br d, *J* = 14.8 Hz, Hb-2), 4.688 (1H, m, H-3), 6.208 (1H, br s, H-4), 3.326 (1H, d, *J* = 15.0 Hz, Ha-6), 3.152 (1H, dd, *J* = 15.0, 1.9 Hz, Hb-6), 2.435 (1H, dd, *J* = 13.8, 5.0 Hz, Ha-7), 2.012 (1H, dd, *J* = 13.8, 11.1 Hz, Hb-7), 4.126 (1H, m, H-8), 1.406 (2H, m, H_2_-9), 1.144–1.353 (22H, m, H_2_-10−H_2_-18, H_2_-23, H_2_-24), 2.097 (4H, m, H_2_-19, H_2_-22), 5.481 (2H, AB type, H-20, H-21), 0.876 (3H, t, *J* = 6.9 Hz, H_3_-25).

*Choerosponin B-(R)-MTPA Ester* (pyridine-*d*_5_, 400 MHz) δ: 3.021 (1H, d, *J* = 14.6 Hz, Ha-2), 2.908 (1H, br d, *J* = 14.6 Hz, Hb-2), 4.770 (1H, m, H-3), 6.273 (1H, br s, H-4), 3.328 (1H, d, *J* = 15.2 Hz, Ha-6), 3.137 (1H, dd, *J* = 15.2, 2.3 Hz, Hb-6), 2.346 (1H, dd, *J* = 14.0, 5.3 Hz, Ha-7), 1.985 (1H, dd, *J* = 14.0, 10.4 Hz, Hb-7), 4.304 (1H, m, H-8), 1.494 (1H, m, Ha-9), 1.385 (1H, m, Hb-9), 1.144-1.353 (22H, m, H_2_-10−H_2_-18, H_2_-23, H_2_-24), 2.096 (4H, m, H_2_-19, H_2_-22), 5.479 (2H, AB type, H-20, H-21), 0.876 (3H, t, *J* = 6.9 Hz, H_3_-25).

### 4.6. Bioassays

#### 4.6.1. Cell Line and Cell Culture

A mouse temperature-sensitive p34^cdc2^ mutant, tsFT210, human cancer HCT-15, HeLa, A2780, MCF-7 cell lines and human umbilical vein endothelial cell ECV304, were used for bioassay. The p34^cdc2^ mutant, tsFT210, HCT-15, HeLa, A2780, MCF-7, ECV304 cells were routinely maintained in RPMI-1640 medium containing 100 µg/mL penicillin and 100 µg/mL streptomycin supplemented with 10% FBS under a humidified atmosphere of 5% CO_2_ and 95% air. The tsFT210 cells were cultured at 32 °C, while the other cells were at 37 °C.

#### 4.6.2. Cell Proliferation Assay

The cell proliferation assay was performed according to the procedure in literature [11].

First 5 μL of sample solutions in MeOH was added, respectively, into each well of the 24-well plate containing the exponentially growing tsFT210 cells at the density of 2 × 10^5^ cell·mL^−1^ in 0.5 mL of fresh medium and the cells were cultured at 32 °C for 17 h. Then the cells were transferred into the 1.5 mL Eppendorf centrifuge tubes, harvested by centrifugation at 3000 rpm for 3 min at 4 °C, washed once with cold phosphate-buffered saline (PBS), and harvested again by centrifugation under the same condition. Then, 150 μl of propidium iodide in water solution (propidium iodide 50 mg/mL, sodium citrate 0.1% and Nonidet p-40 0.2%) was added into each of the tubes and the cells were stained at 4 °C for 30 min. The cells were then subjected to a flow cytometric analysis after dilution with the same volume of PBS and the distribution within the cell cycle was analyzed by WinCycle software (Coulter Co., Hialeah, FL, USA.).

Exponentially growing HCT 15, HeLa, A2780, MCF-7 cells were suspended in fresh medium at the density of 2 × 10^5^ cells·mL^−1^; seeded into 96-well plate and cultured for 24 h under the presence or absence of **1**–**2** at various concentrations and 5-Fu at 100 μg/mL. The cells were fixed with 50 μL of 20% TCA solution for 1 h at 4 °C, washed five times with water and air dried. Then, 50 μL of SRB solution (0.4% in 1% acetic acid) was added and stained at room temperature for 30 min. The unbound dye was removed by washing four times with 1% acetic acid and then air-dried. To each well, 150 μL of Tris buffer solution (10 mM, pH 10.5) was added to dissolve the protein-bound dye and the optical density (OD) of each well was determined on a SPECTRA MAX Plus plate reader at 520 nm. Data from triple wells were taken for each concentration of samples on each plate. The inhibition rates (IR%) for compounds **1**–**2** were calculated using mean OD values from IR% = (OD_control_ − OD_sample_)/OD_control_ × 100%. Then the concentration required for 50% inhibition of the cell growth, IC_50_, was determined using the Bliss method.

#### 4.6.3. Anti-Hypoxia Assay

The anti-hypoxia assay was performed according to our previous procedure [3].

Compounds **1**, **2** and baicalin were dissolved in the RPMI 1640 medium to prepare a solution at 50.0 µg/mL.

Exponentially growing ECV 304 cells were suspended in fresh RPMI 1640 medium at the density of 1 × 10^5^ cells/mL and then seeded into 96-well plates at 150 µL/well. The cells were incubated at 37 °C for 48 h, then discarded the medium and were assigned into normoxic control group, hypoxia control group and hypoxia administration group. Each well of the normoxic control group and hypoxia control group was added 150 µL fresh RPMI 1640 medium, and the hypoxia administration group was 150 µL sample solution. The hypoxia control group and hypoxia administration group were cultured in the atmosphere of 5% CO_2_ and 95% N_2_ for 24 h, while the normal control group was normally cultured for 24 h. Then, 15 µL MTT solution (5 mg/mL in PBS) was added to each well and incubated at 37 °C for 4 h. Then, the MTT solution were discarded, and 150 µL DMSO were added in each well; after the purple material were fully dissolved, the optical density (OD) of each well was determined on a Versa max plate reader at 490 nm. The cell viabilities were calculated using mean values from cell viabilities (%) = OD_hypoxia control_ or OD_sample_/OD_normal control_ × 100%.

#### 4.6.4. Antibacterial Effect Test

*Blastomyces albicans* ATCC10231 and *Staphylococcus aureus* ATCC6538 were used to evaluate the antibacterial activities of compounds **1** and **2**. *Blastomyces albicans* ATCC10231 was cultivated on Sabouraud’s agar medium, and *Staphylococcus aureus* ATCC6538 was cultivated on tryptose soya agar medium. All the compounds were dissolved in methanol to prepare 10 mg/mL test sample solutions. Samples (15 µL) were added to the paper discs (8 mm) and dried for 10 min. Then, the discs were put on the tested plate which contained either *Staphylococcus aureus* or *Blastomyces albicans*. Tested plates were cultured at 28 °C for two days and then the diameter of the inhibition zone was measured.

## 5. Conclusions

Two new cytotoxic bridged-ring ketones, choerosponins A (**1**) and B (**2**), were isolated from the CHCl_3_ extract of the stem bark of *Choerospondias axillaries*. The structures of the two compounds were elucidated by their NMR data, CD spectra and the modified Mosher’s method. Compound **1** has a rare dioxatricyclo skeleton. Among the two compounds, **1** showed moderate cytotoxicity against the tsFT210, HCT-15 and HeLa cell lines, but **2** showed strong cytotoxicity against the five tested cancer cell lines.

## Figures and Tables

**Table 1:** The 600 MHz ^1^H-NMR and 150 MHz ^13^C-NMR data ^a^ for **1** and **2**.

No.		1			2		
		δ_H_ (*J* in Hz)	δc	HMBC(H→C)	δ_H_ (*J* in Hz)	δc	HMBC(H→C)
1			212.1 s			206.0 s	
2	H*a* H*e*	2.57 dd (18.4, 1.4) 2.94 dd (18.4, 3.2)	39.5 t	1, 3, 4 1, 3, 4, 6	2.47 dd (15.8, 2.1) 2.68 ddd (15.8, 3.7, 2.8)	48.1 t	1, 3, 4 1, 3, 4, 6
3		4.57 m	71.3 d	1, 5, 8	4.43 m	72.6 d	1, 2, 4, 5, 8
4		4.79 m	73.4 d	6, 8	4.03 br s	75.2 d	2, 3, 5, 6,7
5	H*a* H*e*	2.22 dd (13.8, 1.8) 1.93 ddd (13.8, 2.8, 1.6)	35.7 t	1, 3, 4, 6, 7 1, 3, 4, 6, 7		70.5 s	
6			74.2 s		2.52 dd (15.5, 1.4) 2.70 dd (15.5, 2.8)	55.5 t	1, 4, 5, 7 1, 2, 4, 5, 7
7	H*a* H*e*	1.94 d (13.3) 1.87 dd (13.3, 1.6)	46.0 t	1, 5, 6, 8, 9 1, 5, 6, 8, 9	1.73 dd (14.2, 11.1) 1.99 ddd (14.2, 5.0, 1.4)	43.7 t	
8			107.6 s		4.25 m	69.4 d	4, 5, 6, 8, 9 4, 5, 6, 8, 9
9	H*a* H*b*	1.72 (2H) m	36.1 t	7, 8, 10, 11	1.38 m 1.24 m	36.8 t	3, 9, 10
10		1.42 (2H) m	23.1 t	8, 9, 11, 12	1.29-1.35 ^b^ m 1.24 m	25.4 t	7, 8, 10, 11 7, 8, 10, 11
11		1.24–1.29 ^b^ m	29.2 t	9, 10, 12, 13	1.22–1.29 ^b^ m	29.5–29.7 ^b^ t	9, 10, 12, 13
12		1.24–1.29 ^b^ m	29.3 t	10, 11, 13, 14	1.22–1.29 ^b^ m	29.5–29.7 ^b^ t	10, 11, 13, 14
13–16		1.24–1.29 ^b^ m	29.3-29.5 ^b^ t	11,12,14–16,17,18	1.22–1.29 ^b^ m	29.5–29.7 ^b^ t	11, 12~17,18,
17		1.24–1.29 ^b^ m	29.3-29.5 ^b^ t	15, 16, 18, 19	1.22–1.29 ^b^ m	29.6 t	15, 16, 18, 19
18		1.29–1.35 ^b^ m	29.6 t	16, 17, 19, 20	1.29–1.35 ^b^ m	29.3 t	16, 17, 19, 20
19		2.02 (2H) m	26.9 ^c^ t	17, 18, 20, 21	2.02 (2H) m	26.9 ^c^ t	17, 18, 20, 21
20		5.35 m	129.9 ^d^ d	18, 19, 22	5.35 m	129.9 ^d^ d	19, 22
21		5.35 m	129.8 ^d^ d	19, 22	5.35 m	129.8 ^d^ d	19, 22
22		2.02 (2H) m	27.2 ^c^ t	20, 21, 23, 24	2.02 (2H) m	27.2 ^c^ t	20, 21, 23, 24
23		1.29–1.35 ^b^ m	22.3 t	21, 22, 24, 25	1.29–1.35 ^b^ m	22.3 t	21, 22, 24
24		1.29–1.35 ^b^ m	31.9 t	22, 23, 25	1.29–1.35 ^b^ m	31.9 t	22, 23
25		0.90 (3H) t (7.1)	14.0 q	23, 24	0.90 (3H) t (7.1)	14.0 q	23, 24
4-OH					2.84 br s		3
5-OH					2.36 br s		
6-OH		3.77 s		1, 5, 6			

^a^ Data of **1** and **2** in CDCl_3_ solution. Signal assignments were based on the results of DEPT, ^1^H-^1^H COSY, HMQC, HMBC and difference NOE experiments. ^b^ The signals could not be assigned exactly because of the signal overlapping. ^c^ and ^d^ Signal assignments may be interchanged between two signals with the same superscript.

**Table 2:** The inhibiting effect of **1** on HCT-15 and HeLa cells.

Cells	IR%					IC_50_ (µM)
	200 µM	100 µM	10 µM	1 µM	0.1 µM	
HCT-15	73.3%	47.3%	28.2%	38.1%	38.0%	104.3
HeLa	62.1%	40.4%	35.1%	34.1%	32.3%	144.0

**Table 3:** The inhibiting effect of **2** on HCT-15, HeLa, A2780 and MCF-7 cells.

Cells	IR%					IC_50_ (µM)
	200 µM	100 µM	10 µM	1 µM	0.1 µM	
HCT-15	90.9%	86.5%	68.7%	22.3%	NA	3.3
HeLa	64.6%	86.5%	42.5%	24.6%	26.4%	11.1
A2780	70.9%	73.1%	15.5%	NA	NA	13.2
MCF-7	68.3%	68.0%	10.2%	8.1%	NA	71.1

NA: No activity.

**Table 4:** Anti-hypoxia effects of **1** and **2** on anoxic tested cells (50 µg/mL).

Samples	Tested Cells	Cell Viabilities (Mean Value ± SD%, *n* = 10)	
		Control Group	Test Group
**1**	ECV304	22.6 ± 0.1	14.0 ± 0.7
**2**	ECV304	22.6 ± 0.1	1.0 ± 0.5
Baicalin *	ECV304	22.6 ± 0.1	51.7 ± 1.7

Baicalin * was used as positive control.

## Supplementary Materials

Supplementary materials are available online. Figures S1–S22: UV, ESI-MS, NMR, and IR spectra of **1** and **2**; Figure S23: ^1^H-NMR spectrum of the *S* and *R* MTPA esters of **2**; Figure S24: Flow cytometric histogram of tsFT210 cells treated with **1** and **2**; Tables S1 and S2: 600 MHz ^1^H and 150 MHz ^13^C-NMR Data for **1** and **2** in CDCl_3_.
