# Supplementary material for: Choerosponins A and B, Two New Cytotoxic Bridged-Ring Ketones and the Determination of Their Absolute Configurations
Source: Molecules. 2017 Mar 27;22(4):531. doi: 10.3390/molecules22040531 (PMC6154574; doi:10.3390/molecules22040531)
Supplement: Supplementary file 1 [file molecules-22-00531-s001.pdf]

Supplementary Information

Contents

Contents	page
**Figure S1**. UV spectrum and of **1** in MeOH	S2
**Figure S2**. Positive ESI-MS spectrum of **1**	S2
**Figure S3**. 600 MHz ^{1}H NMR spectra of **1** in $CDCl_3$	S3
**Figure S4.** 150 MHz ^{13}C NMR spectra of **1** in $CDCl_3$	S3
**Figure S5**. DEPT spectrum of **1** in $CDCl_3$	S4
**Figure S6**. HMQC spectrum of **1** in $CDCl_3$	S4
**Figure S7**. ^{1}H-^{1}H COSY spectrum of **1** in $CDCl_3$	S5
**Figure S8**. HMBC spectrum of **1** in $CDCl_3$	S5
**Figure S9**. NOE difference experiment spectra of **1** in $CDCl_3$	S6
**Figure S10**. IR spectrum of **1**	S7
**Figure S11**. CD spectrum of **1** in MeOH	S7
**Figure S12**. UV spectrum and of **2** in MeOH	S8
**Figure S13**. Positive ESI-MS spectrum of **2**	S8
**Figure S14**. 600 MHz ^{1}H spectra of **2** in $CDCl_3$	S9
**Figure S15**. 150 MHz ^{13}C NMR spectra of **2** in $CDCl_3$	S9
**Figure S16**. DEPT spectrum of **2** in $CDCl_3$	S10
**Figure S17**. HMQC spectrum of **2** in $CDCl_3$	S10
**Figure S18**. ^{1}H-^{1}H COSY spectrum of **2** in $CDCl_3$	S11
**Figure S19**. HMBC spectrum of **2** in $CDCl_3$	S11
**Figure S20**. NOE difference experiment spectra of **2** in $CDCl_3$	S12
**Figure S21**. IR spectrum of **2**	S13
**Figure S22**. CD spectrum of **2** in MeOH	S14
**Figure S23**. ^{1}H NMR spectrum of the *S* and *R* MTPA esters of **2**	S14
**Figure S24**. Flow Cytometric Histogram of tsFT210 Cells Treated with **1** and **2**	S15
**Table S1**. 600 MHz ^{1}H and 150 MHz ^{13}C NMR Data for**1** in $CDCl_3$	S16
**Table S2**. 600 MHz ^{1}H and 150 MHz ^{13}C NMR Data for**2** in $CDCl_3$	S17

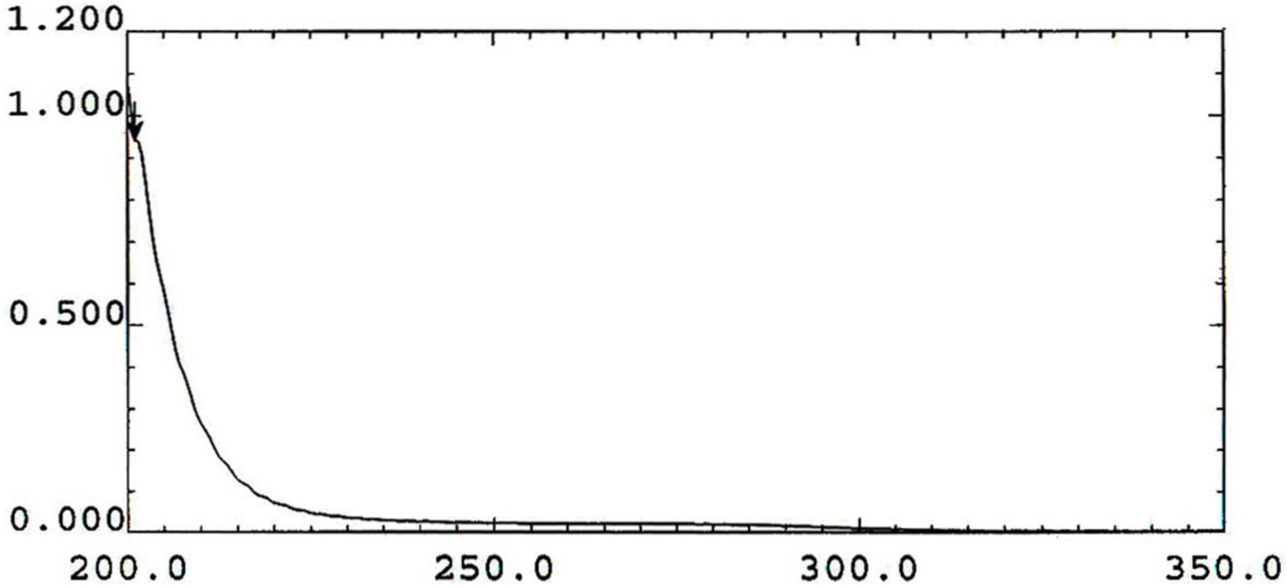

Figure S1. UV spectrum of **1** in MeOH

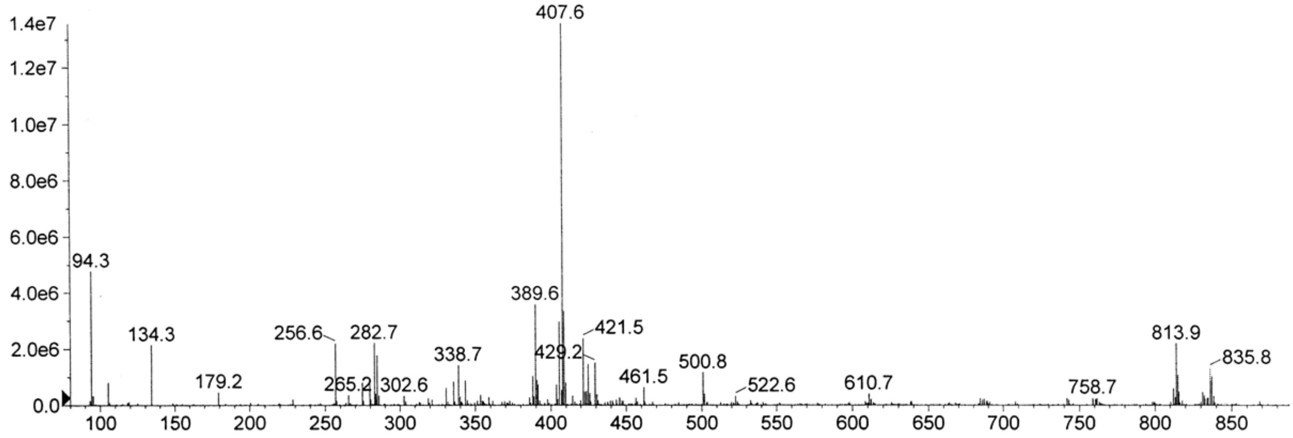

Figure S2. Positive ESI-MS spectrum of **1**

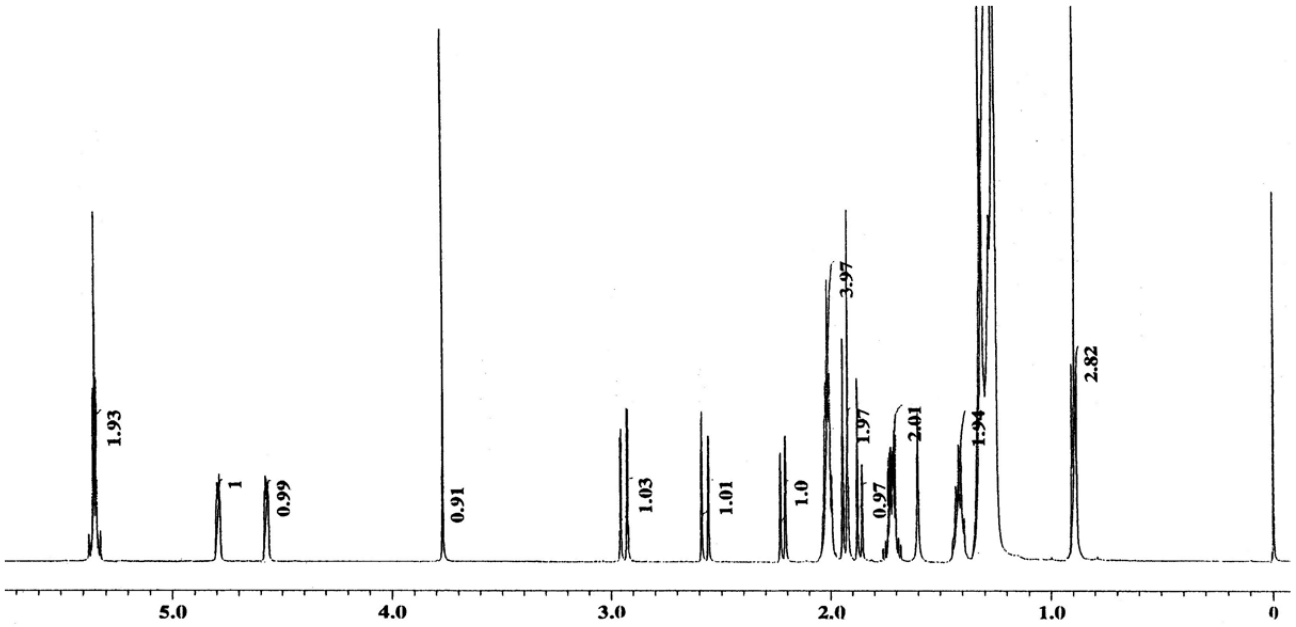

Figure S3. 600 MHz ^{1}H NMR spectrum of **1** in $CDCl_3$

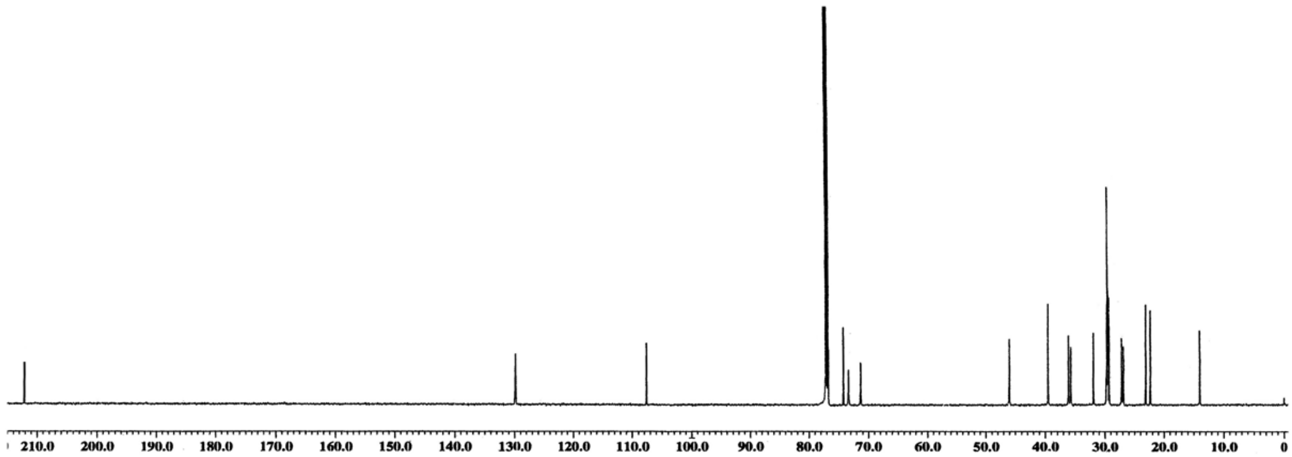

Figure S4. 150 MHz ^{13}C NMR spectrum of **1** in $CDCl_3$

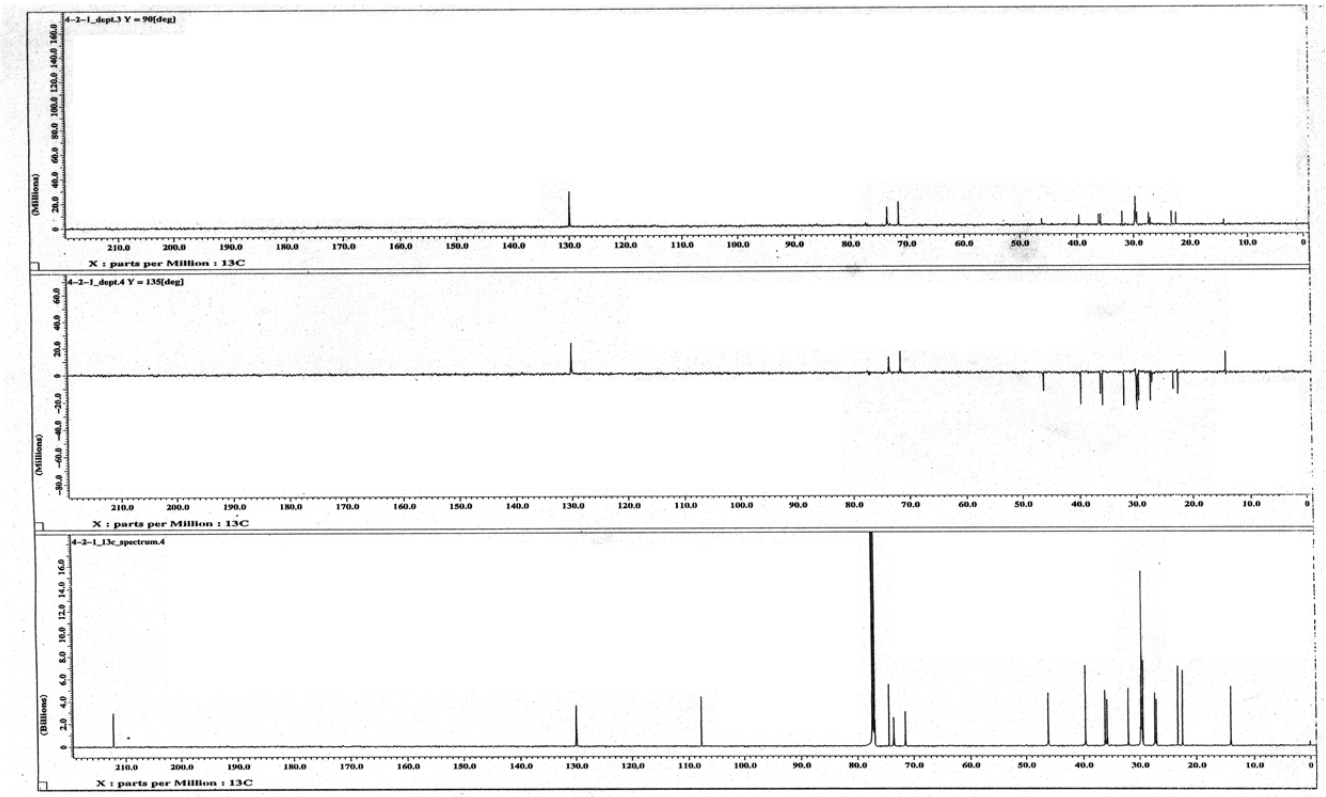

Figure S5. DEPT spectrum of **1** in $CDCl_3$

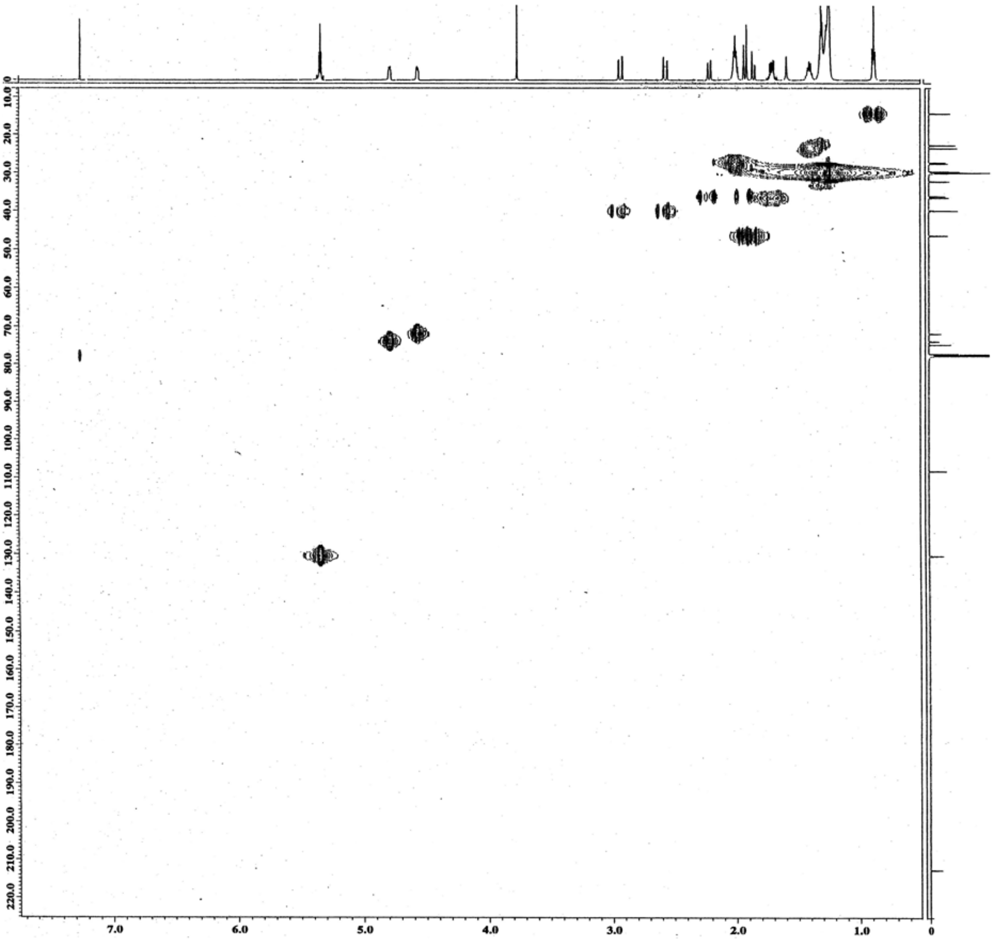

Figure S6. HMQC spectrum of **1** in $CDCl_3$

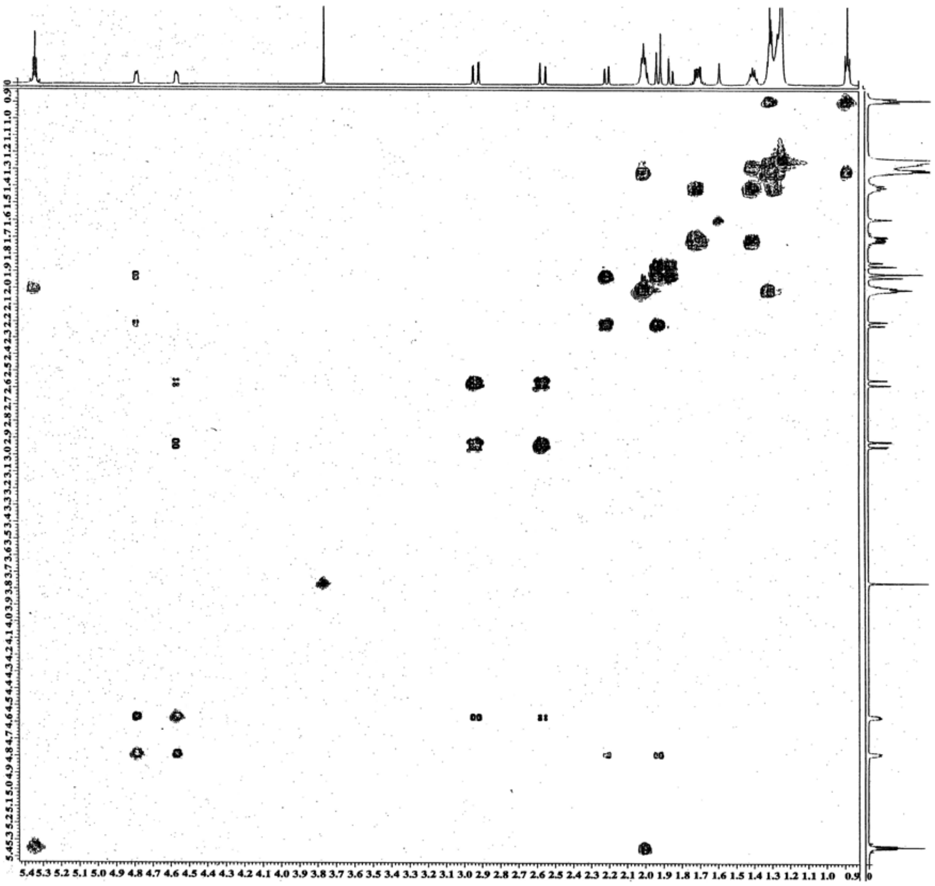

Figure S7. ^1H-^1H COSY spectrum of **1** in $CDCl_3$

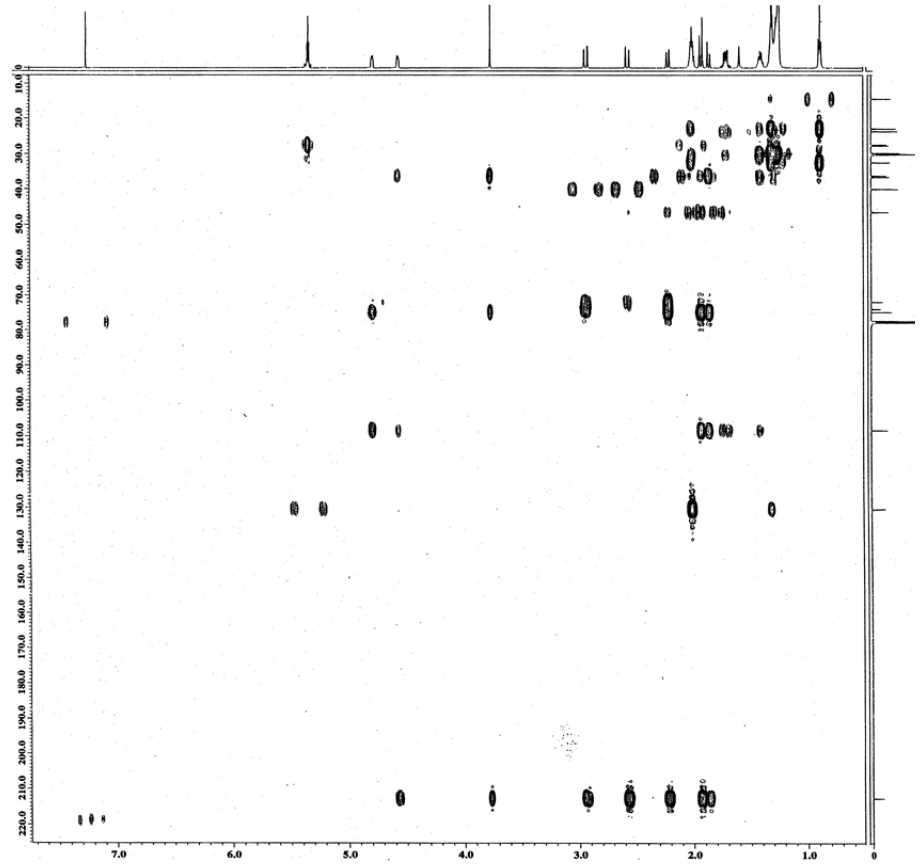

Figure S8. HMBC spectrum of **1** in $CDCl_3$

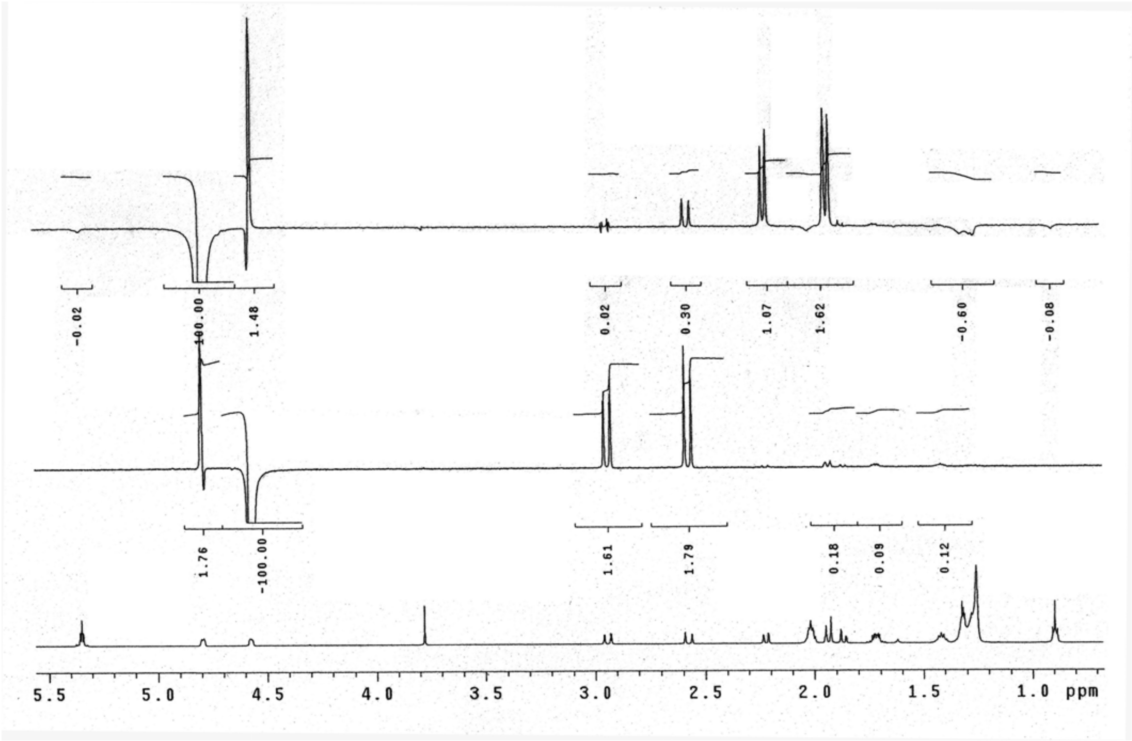

Figure S9 (A). NOE difference experiment spectrum of **1** in $CDCl_3$

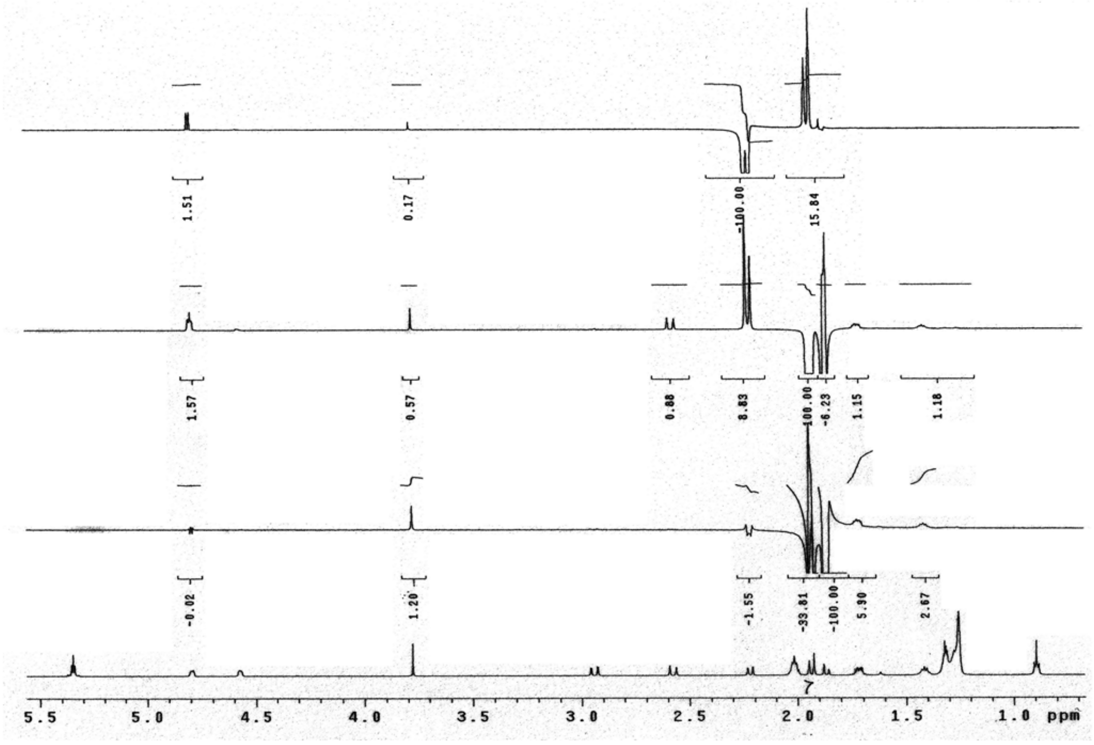

Figure S9 (B). NOE difference experiment spectrum of **1** in $CDCl_3$

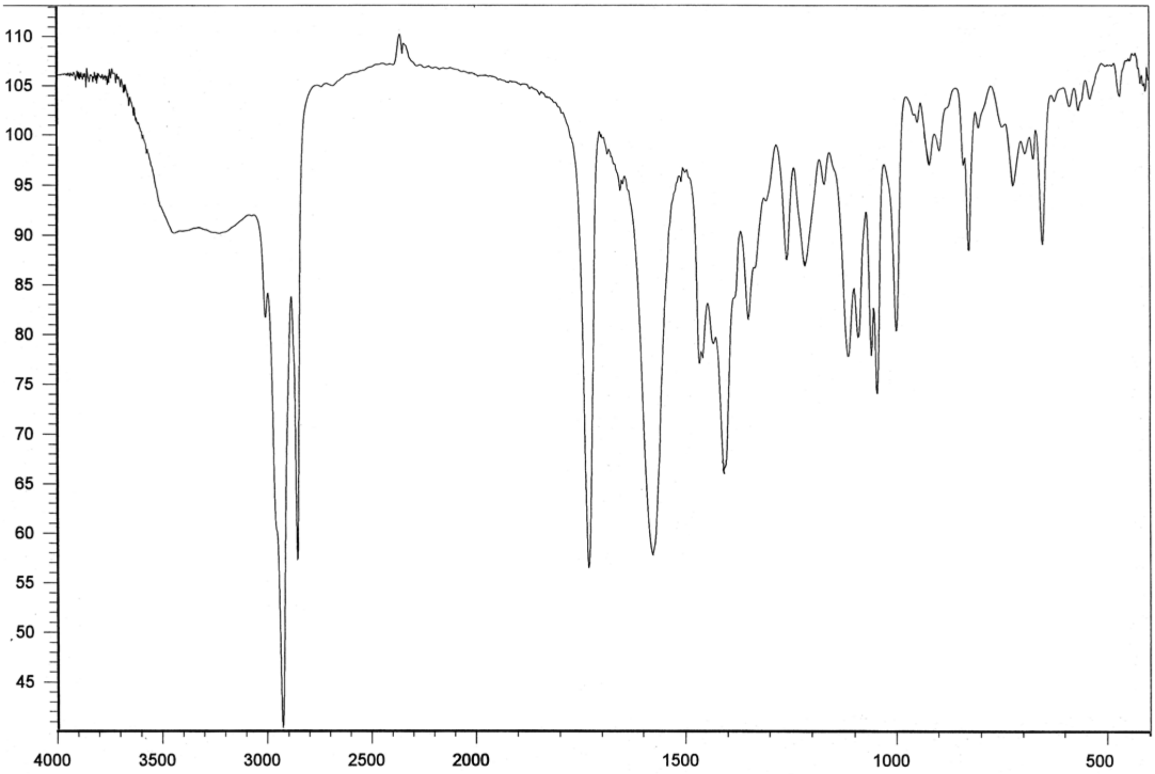

Figure S10. IR spectrum of **1** (in KBr)

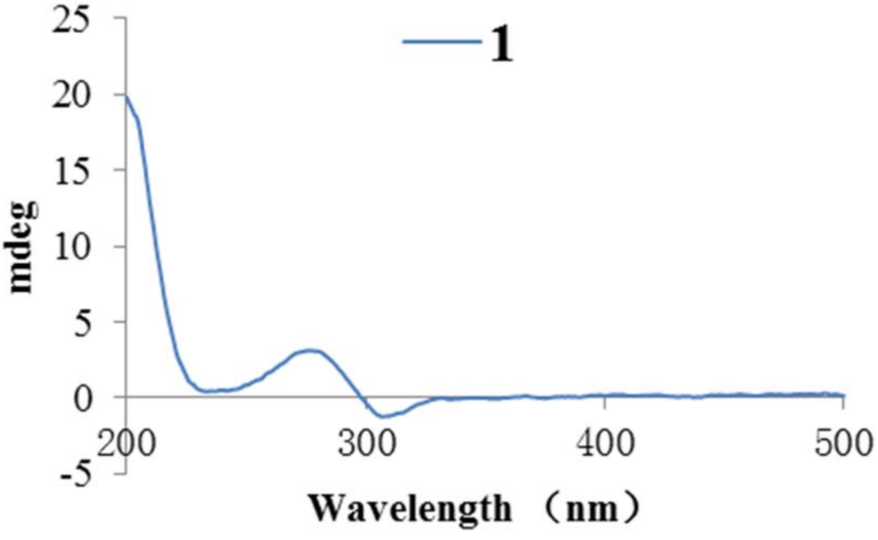

Figure S11. CD spectrum of **1** in CH_3OH

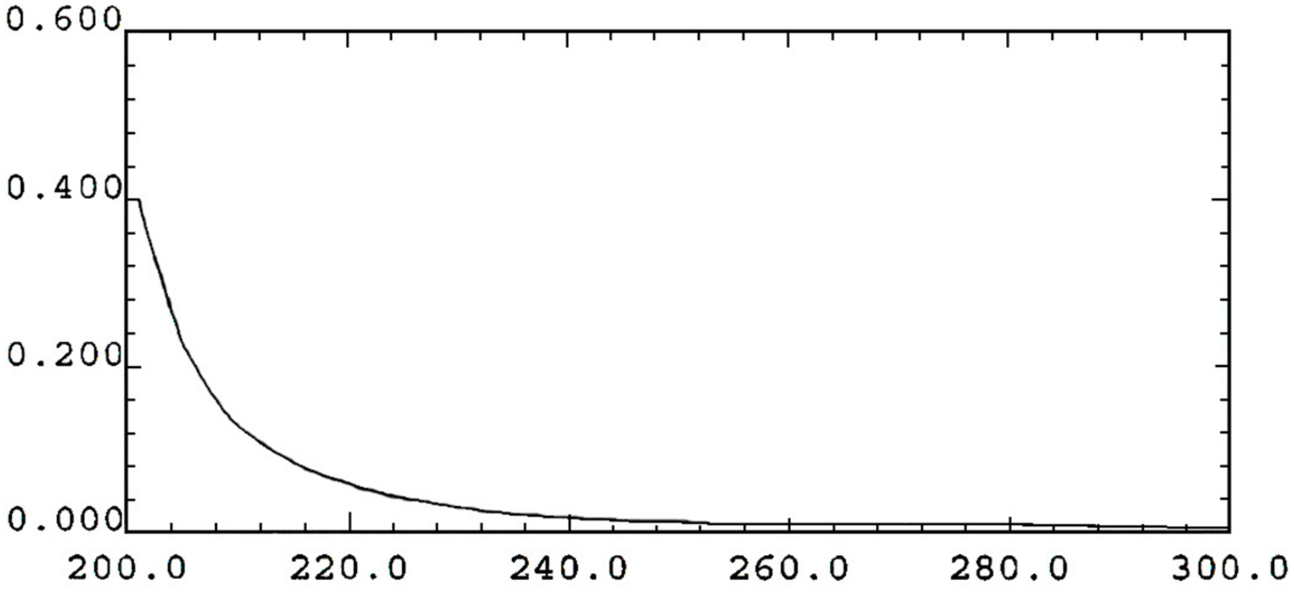

Figure S12. UV spectrum of **2** in MeOH

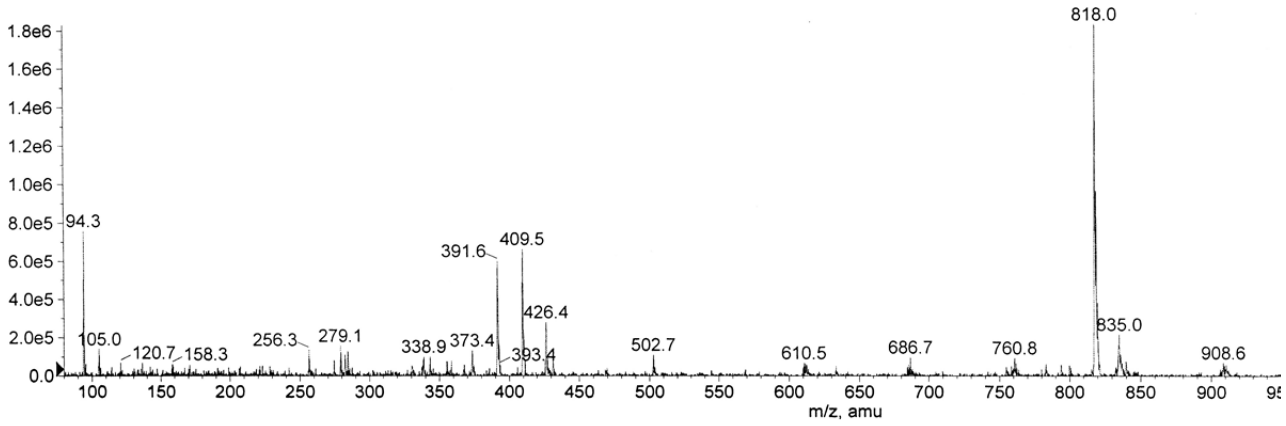

Figure S13. Positive ESI-MS spectrum of **2**

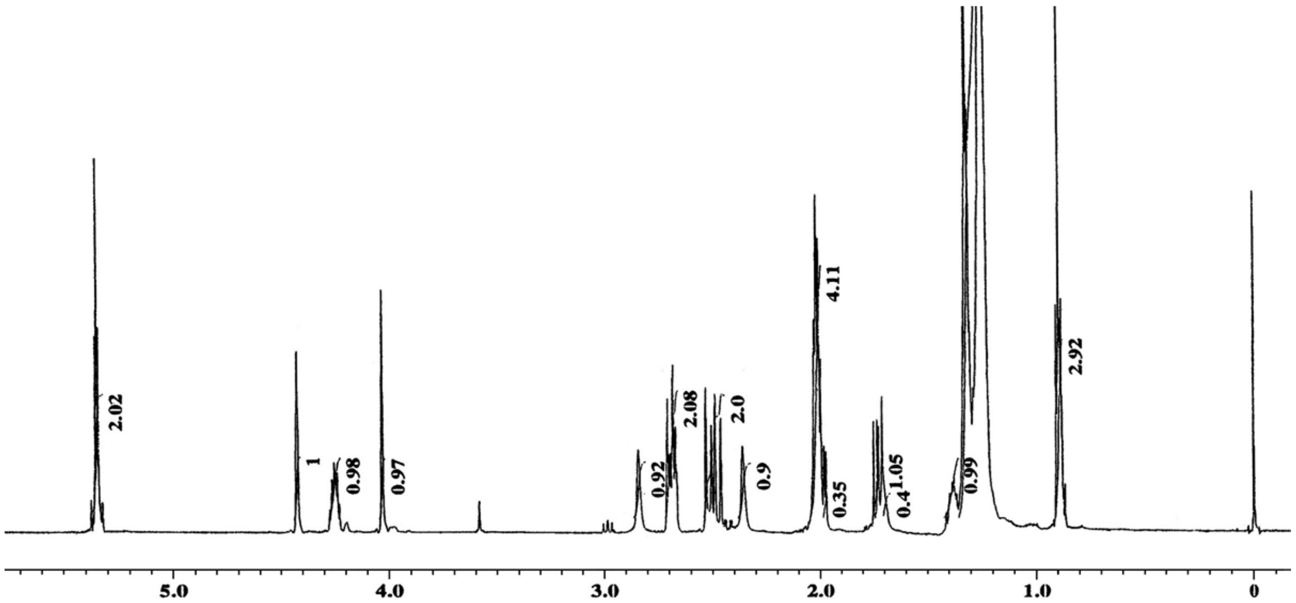

Figure S14. 600 MHz ^{1}H NMR spectrum of **2** in $CDCl_3$

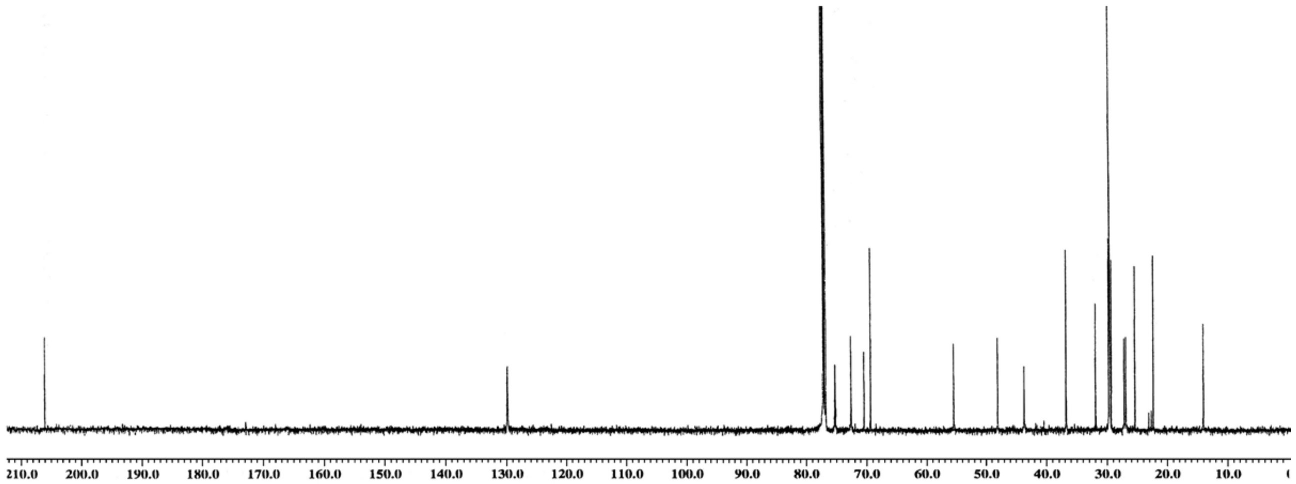

Figure S15. 150 MHz ^{13}C NMR spectrum of **2** in $CDCl_3$

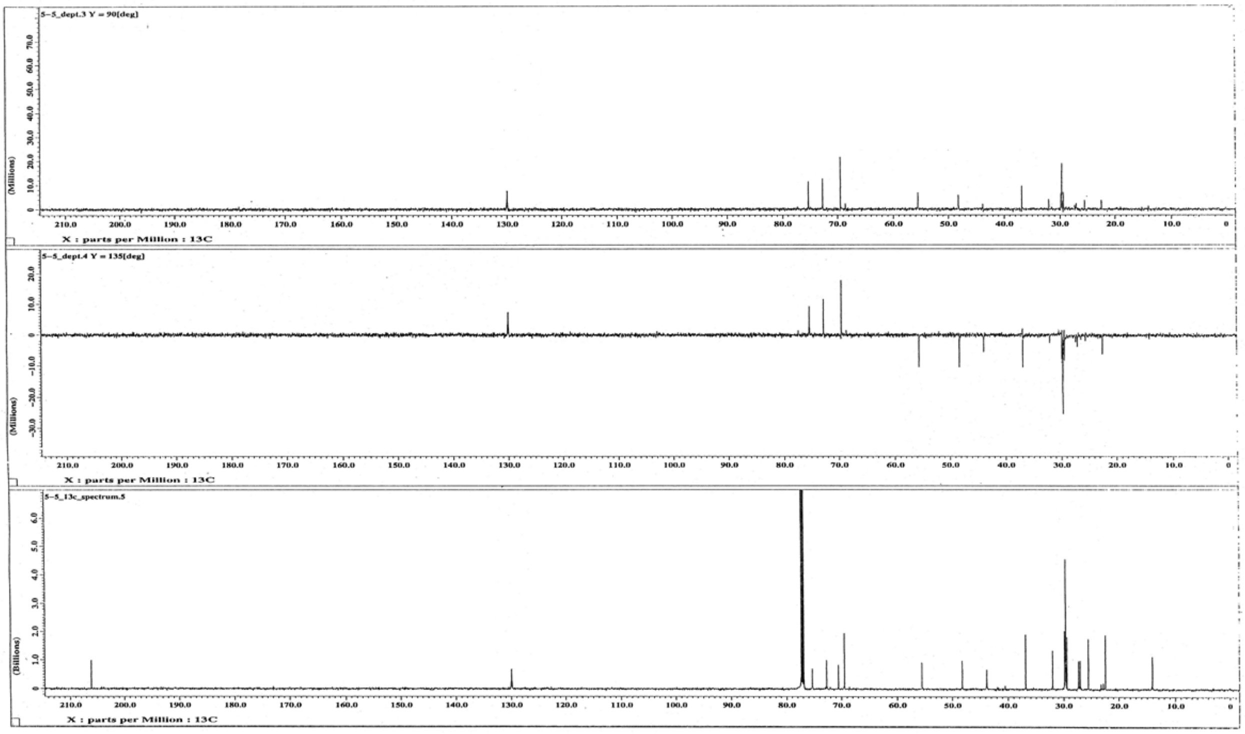

Figure S16. DEPT spectrum of **2** in $CDCl_3$

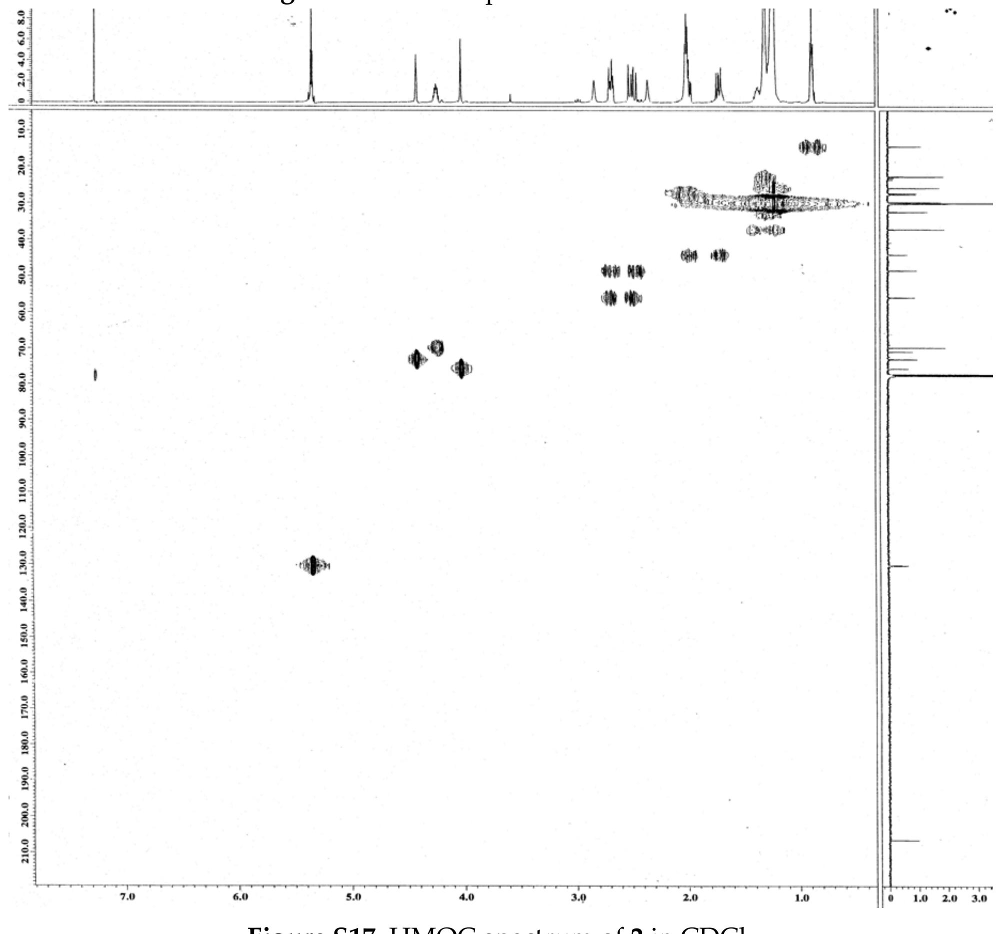

Figure S17. HMQC spectrum of **2** in $CDCl_3$

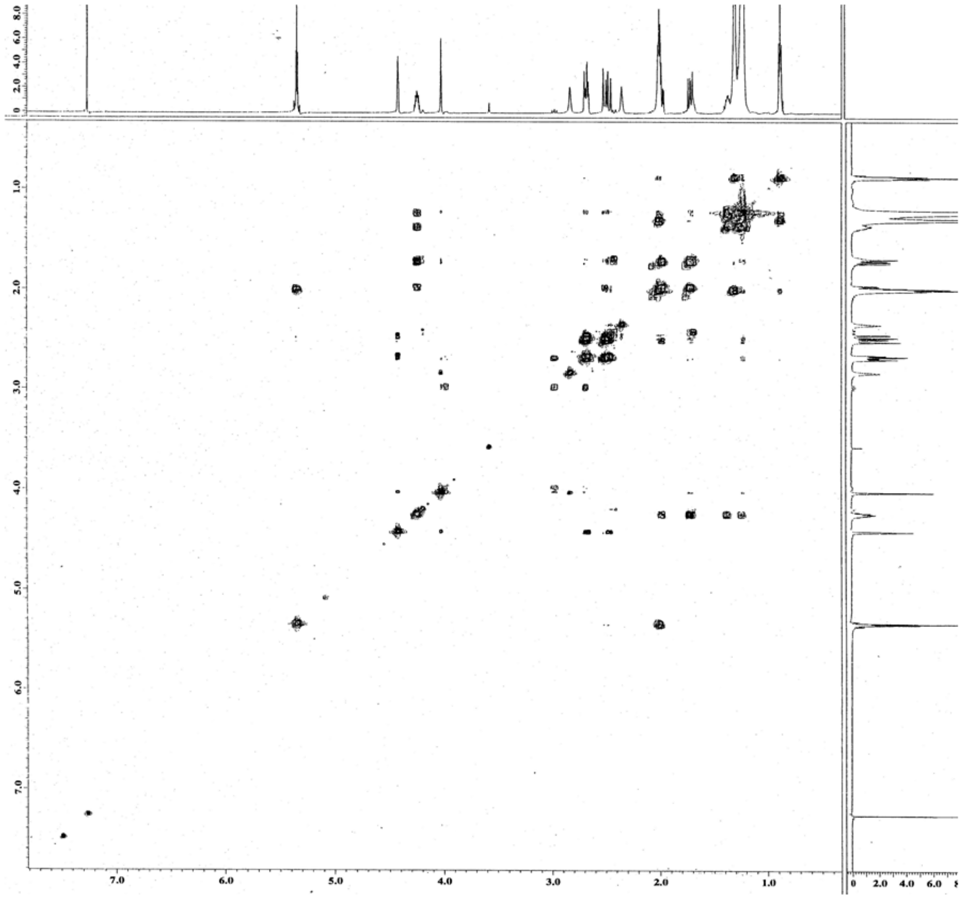

Figure S18. ^{1}H-^{1}H COSY spectrum of **2** in $CDCl_3$

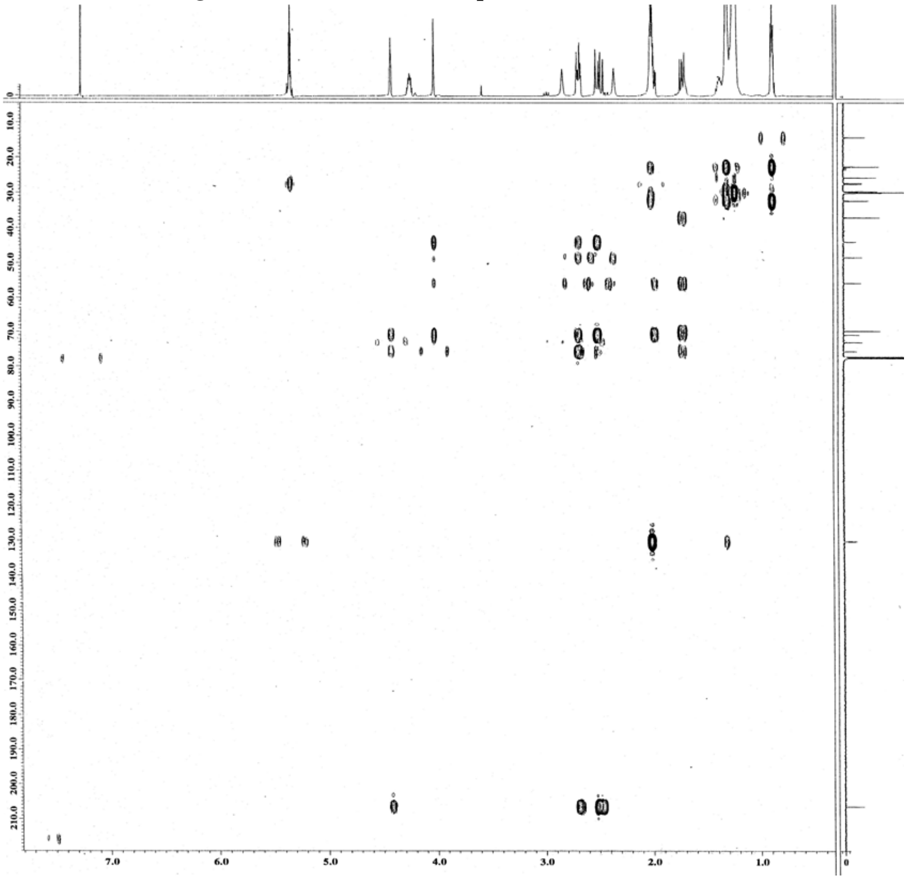

Figure S19. HMBC spectrum of **2** in $CDCl_3$

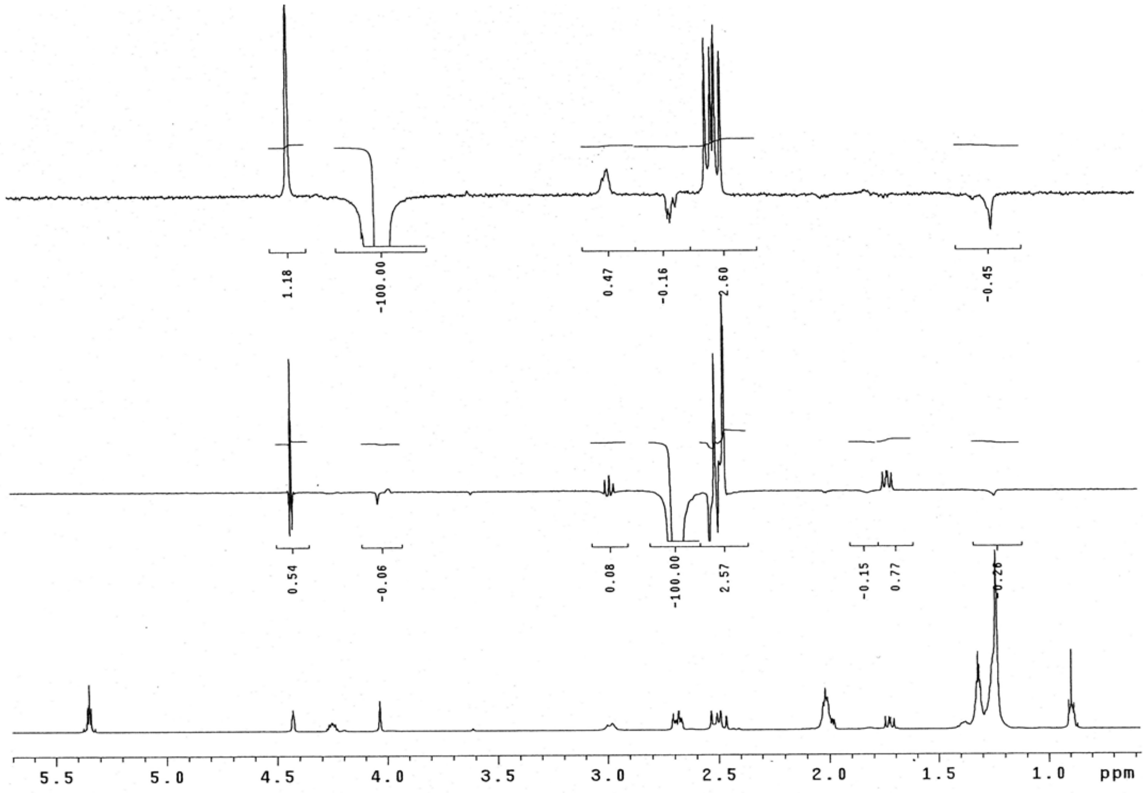

Figure S20 (A). NOE difference experiment spectrum of **2** in $CDCl_3$

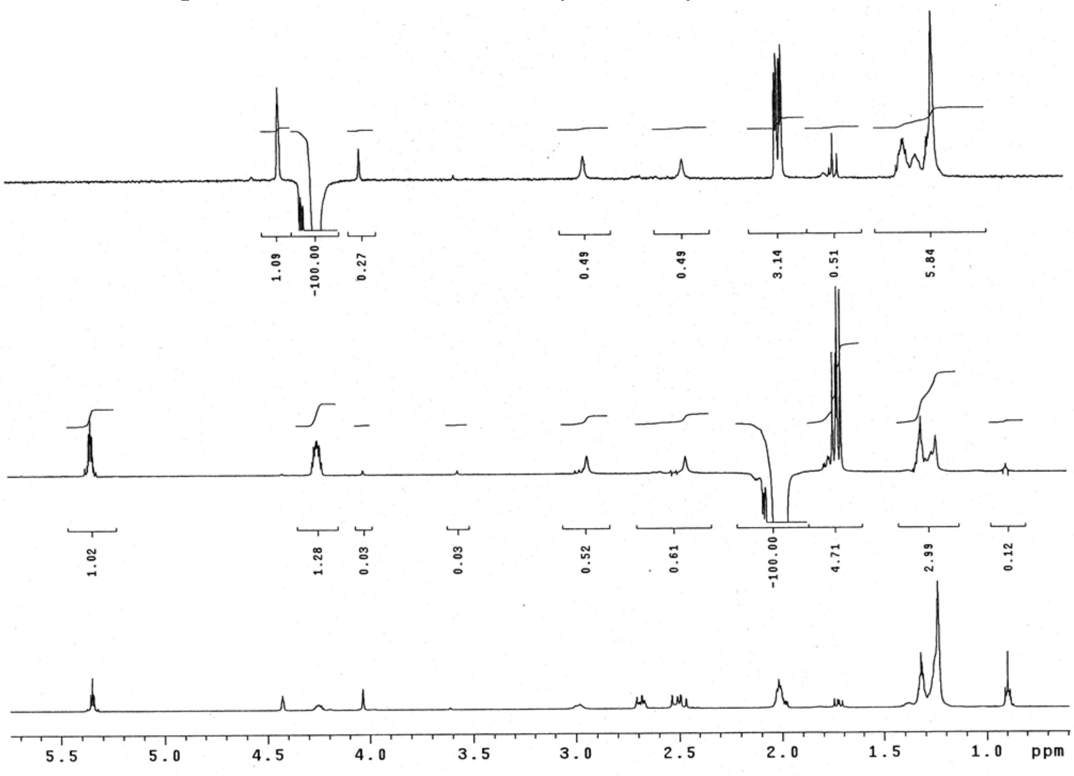

Figure S20 (B). NOE difference experiment spectrum of **2** in $CDCl_3$

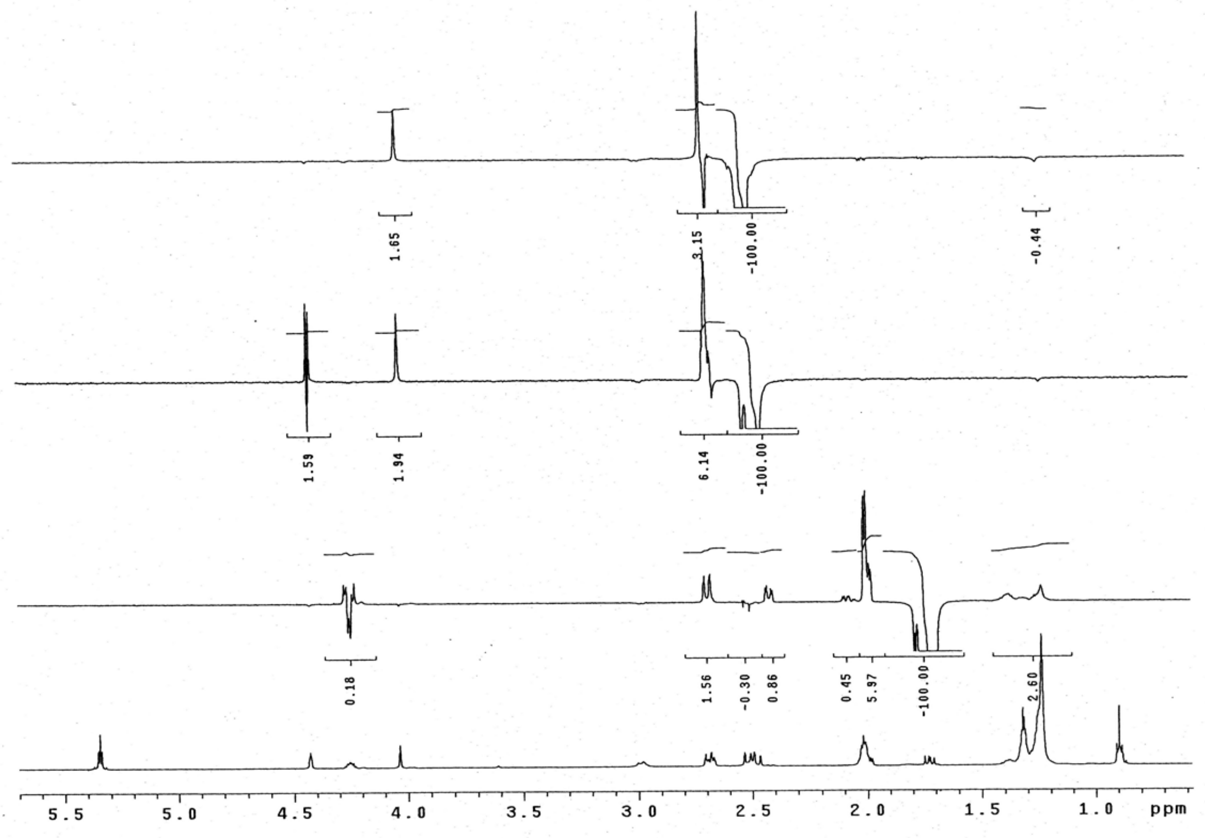

Figure S20 (C). NOE difference experiment spectrum of **2** in $CDCl_3$

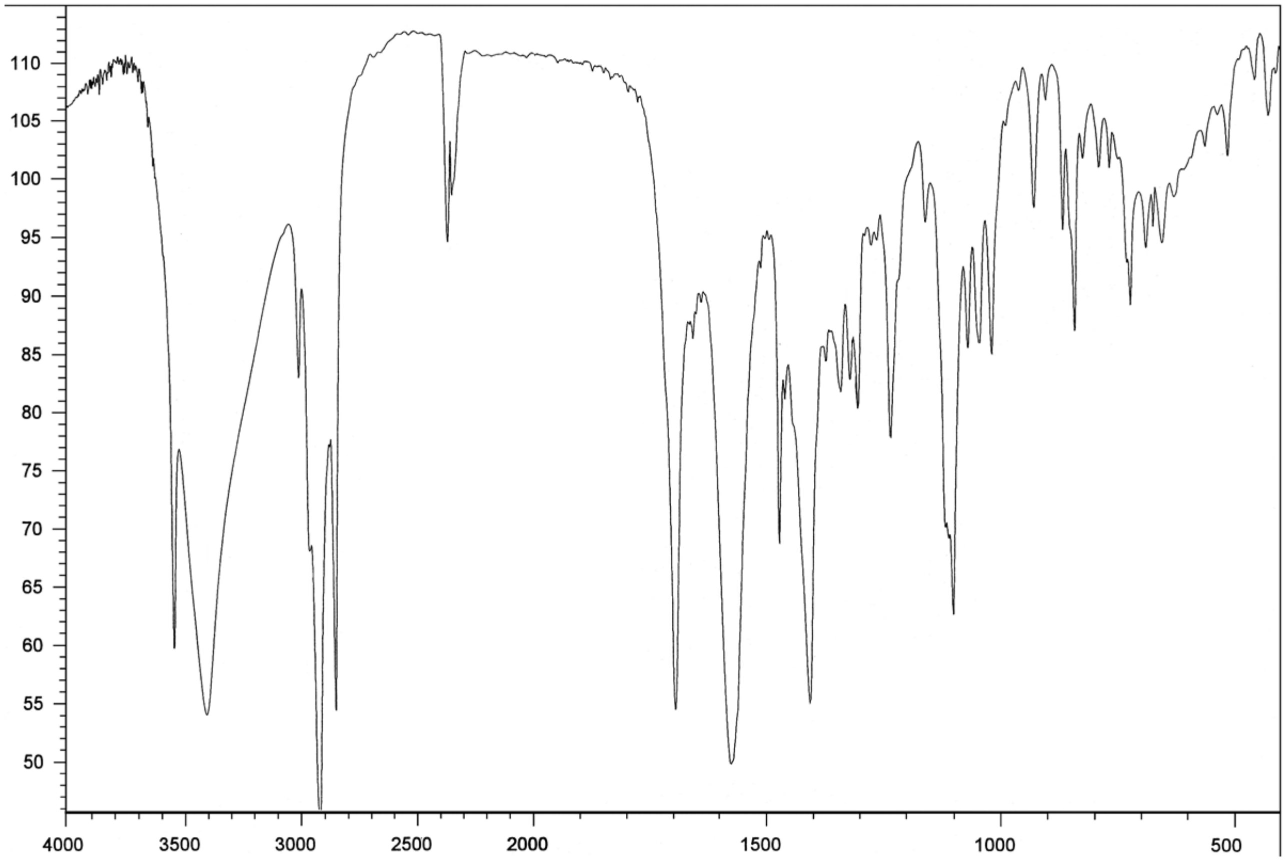

Figure S21. IR spectrum of **2** (in KBr)

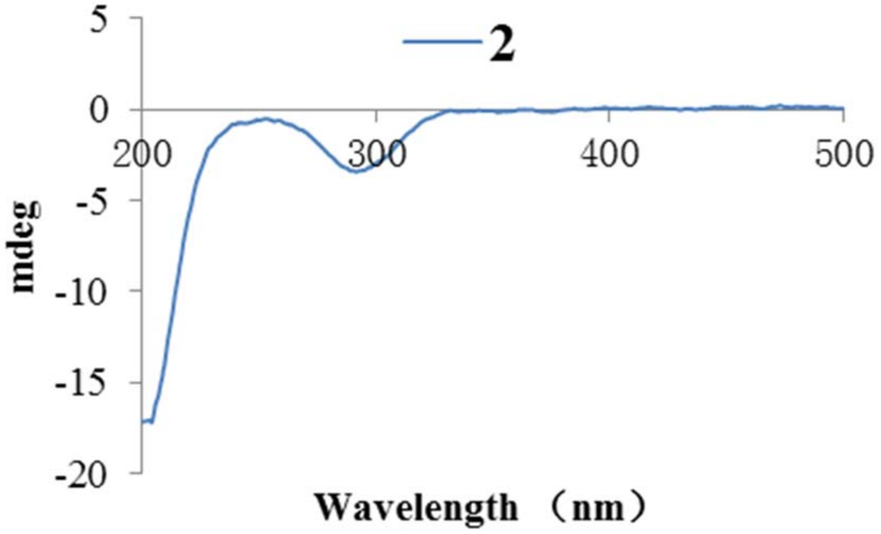

Figure S22. CD spectrum of **2** in MeOH

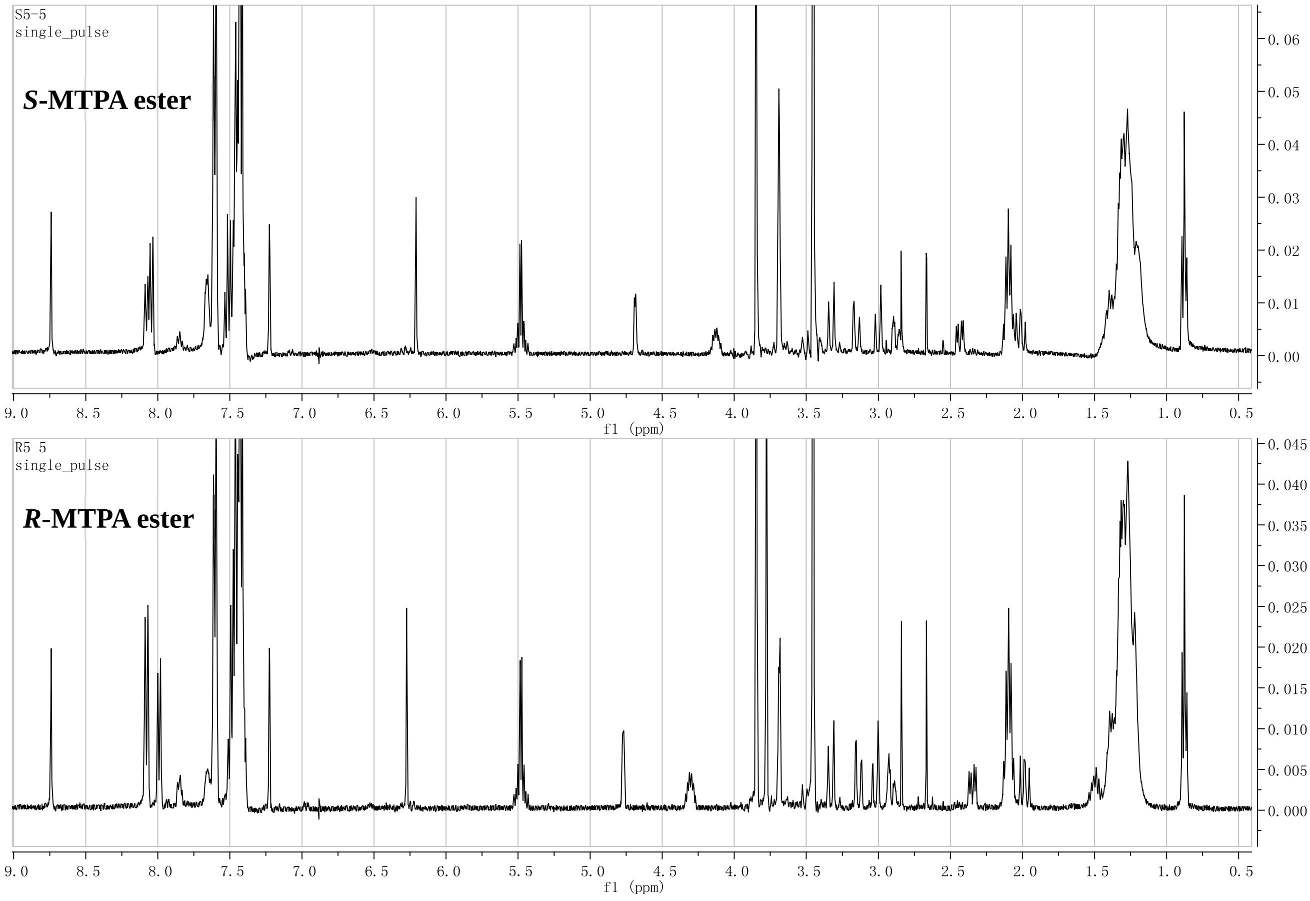

Figure S23. 400 MHz ^{1}H NMR spectrum of the *S* and *R* MTPA esters of **2** in pyridine-d_5

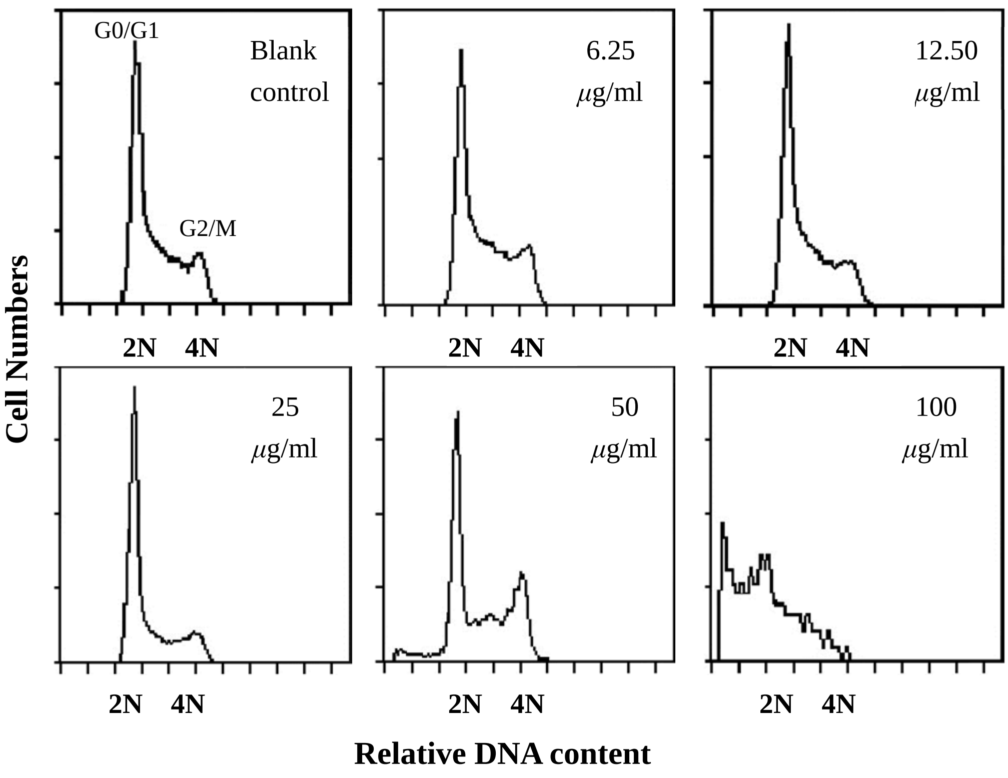

Figure S24 (A). Flow Cytometric Histogram of tsFT210 Cells Treated with **1**

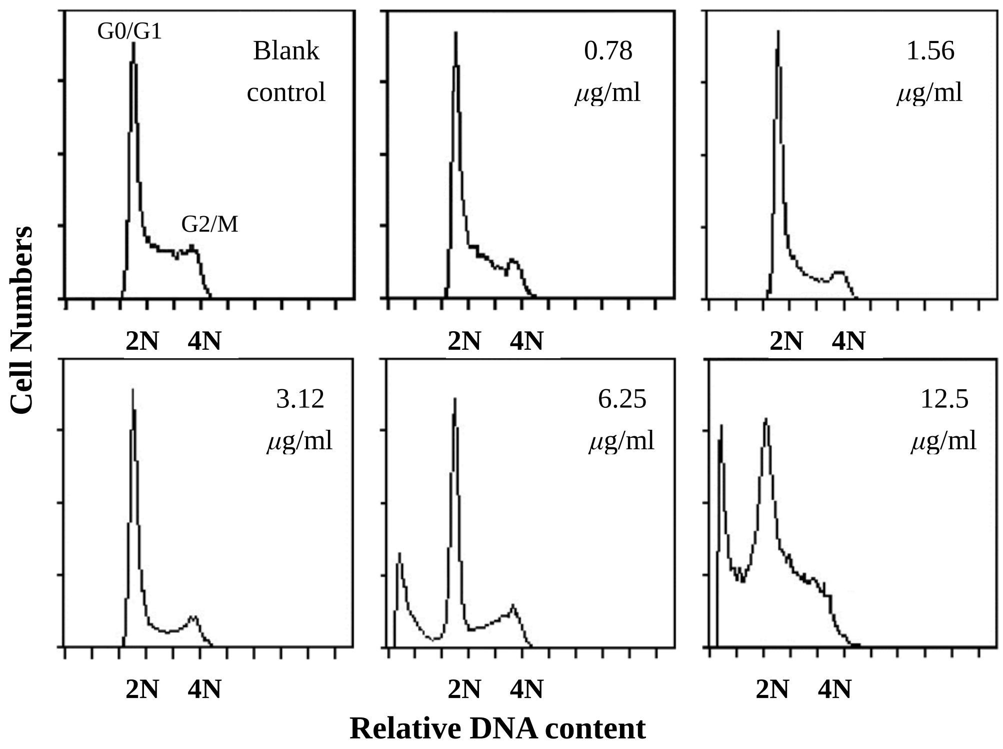

Figure S24 (B).Flow Cytometric Histogram of tsFT210 Cells Treated with **2**

Table S1. 600 MHz ^{1}H and 150 MHz ^{13}C NMR Data for **1** in $CDCl_3$ [a)]

No.		δ_H (*J* in Hz)	COSY[b)]	NOE's[c)]	δ_C	HMBC[d)]
1					212.10 s	
2	H*a*	2.57 dd (18.4, 1.4)	H-3		39.53 t	C-1, C-3, C-4
	H*e*	2.94 dd (18.4, 3.2)	H-3			C-1, C-3, C-4, C-6
3		4.57 m	H*a*-2, H*e*-2, H-4	H*a*-2, H*e*-2, H-4	71.34 d	C-1, C-5, C-8
4		4.79 m	H-3, H*a*-5, H*e*-5	H*a*-2, H-3, H*a*-5, H*e*-5	73.38 d	C-6, C-8
5	H*a*	2.22 dd (13.8, 1.8)	H-4, H*e*-5	H-4, H*e*-5, 6-O<u>H</u>, H*a*-7	35.73 t	C-1, C-3, C-4, C-6, C-7
	H*e*	1.930 dd (13.8, 2.8, 1.6)	H-4, H*a*-5, H*e*-7[e)]	H*a*-2, H-4, H*a*-5, 6-O<u>H</u>		C-1, C-3, C-4, C-6, C-7
6					74.22 s	
7	H*a*	1.935 d (13.3)	H*e*-7	H*a*-5, 6-O<u>H</u>, H*e*-7, H-9, H-10	46.02 t	C-1, C-5, C-6, C-8, C-9
	H*e*	1.87 dd (13.3, 1.6)	H*e*-5[e)], H*a*-7	6-O<u>H</u>, H*a*-7, H-9, H-10		C-1, C-5, C-6, C-8, C-9
8					107.63 s	
9		1.72 (2H) m	H-10		36.10 t	C-7, C-8, C-10, C-11
10		1.42 (2H) m	H-9, H-11		23.13 t	C-8, C-9, C-11, C-12
11		1.24-1.29[f)] m			29.67 t	C-9, C-10, C-12, C-13
12		1.24-1.29[f)] m			29.28 t	C-10, C-11, C-13, C-14
13-17		1.24-1.29[f)] m			29.3-29.5[f)] t	C-11,12,14-16, C-18,19
18		1.29-1.35[f)] m	H-19		29.58 t	C-16, C-17, C-19, C-20
19		2.02 (2H) m	H-18, H-20		26.88[g)] t	C-17, C-18, C-20, C-21
20		5.35 AB type	H-19, H-21		129.88[h)] d	C-18, C-19, C-22
21		5.35 AB type	H-20, H-22		129.82[h)] d	C-19, C-22
22		2.02 (2H) m	H-21, H-23		27.17[g)] t	C-20, C-21, C-23, C-24
23		1.29-1.35[f)] m	H-22, H-24		22.32 t	C-21, C-22, C-24, C-25
24		1.29-1.35[f)] m	H-23, H-25		31.93 t	C-22, C-23, C-25
25		0.90 (3H) t (7.1)	H-24		13.98 q	C-23, C-24
6-O<u>H</u>		3.77 s			———	C-1, C-5, C-6

a) Signal assignments were based on the results of DEPT, PFG ^{1}H-^{1}H COSY, PFG HMQC, PFG HMBC and difference NOE experiments. b) Numbers in the column indicate the protons that correlated with the proton on the line in the PFG ^{1}H-^{1}H COSY. c) Numbers in the column indicate the protons at which NOE's were detected in the difference NOE experiment under irradiation at the proton on the line. d) Numbers in the column indicate the carbons that showed HMBC correlations with the proton on the line in the PFG HMBC spectrum. e) The W-form long-range correlation was detected between H-5*e* and H-7*e* in the PFG ^{1}H-^{1}H COSY. f) The signal could not be assigned exactly because of the signal overlapping. g) and h) Signal assignments may be interchanged between two signals with the same superscript.

Table S2. 600 MHz ^{1}H and 150 MHz ^{13}C NMR Data for **2** in $CDCl_3$[a]

No.		δ$_H$ (*J* in Hz)	COSY[b]	NOE's[c]	δ$_C$	HMBC[d]
1					205.99 s	
2	H*a*	2.47 dd (15.8, 2.1)	H*e*-2, H-3	H*e*-2, H-3, H-4	48.08 t	C-1, C-3, C-4
	H*e*	2.68 ddd (15.8, 3.7, 2.8)	H*a*-2, H-3, H*e*-6[e]	H*a*-2, H-3		C-1, C-3, C-4, C-6
3		4.43 m	H*a*-2, H*e*-2, H-4		72.59 d	C-1, C-2, C-4, C-5, C-8
4		4.03 br s	H-3, 4-OH	H*a*-2[f], H-3, 4-OH, H*a*-6[f]	75.20 d	C-2, C-3, C-5, C-6, C-7
5					70.45 s	
6	H*a*	2.52 dd (15.5, 1.4)	H*e*-6, H*e*-7[g]	H-4, H*e*-6	55.48 t	C-1, C-4, C-5, C-7
	H*e*	2.70 dd (15.5, 2.8)	H*e*-2[e], H*a*-6	H*a*-6, H*a*-7		C-1, C-2, C-4, C-5, C-7
7	H*a*	1.73 dd (14.2, 11.1)	H*e*-7, H-8	H*e*-6, H*e*-7, Ha-9, Hb-9	43.68 t	C-4, C-5, C-6, C-8, C-9
	H*e*	1.99 ddd (14.2, 5.0, 1.4)	H*a*-6[g], H*a*-7, H-8	4-OH, 5-OH, H*a*-7, H-8, H_2-10		C-4, C-5, C-6, C-8, C-9
8		4.25 m	H*a*-7, H*e*-7, Ha-9, Hb-9	H-3, 4-OH, 5-OH, H*e*-7, H_2-9	69.35 d	C-3, C-9, C-10
9	Ha	1.38 m	H-8, Hb-9, H_2-10		36.77 t	C-7, C-8, C-10, C-11
	Hb	1.24 m	H-8, Ha-9, H_2-10			C-7, C-8, C-10, C-11
10	Ha	1.29-1.35[h] m	Ha-9, Hb-9, Hb-10, H-11		25.38 t	C-9, C-11, C-12
	Hb	1.24 m	Ha-9, H-11			C-11, C-12
11-16		1.22-1.29[i] m	H-10, H-17		29.5-29.7[j]t	C10, C-12~C-17,18,19
17		1.22-1.29[i] m	H-16, H-18		29.75 t	C-15, C-16, C-18, C-19
18		1.29-1.35[h] m	H-17, H-19		29.29 t	C-16, C-17, C-19, C-20
19		2.02 (2H) m	H-18, H-20		26.88[k] t	C-17, C-18, C-20, C-21
20		5.35 AB type	H-19, H-21		129.90[l] d	C-19, C-22
21		5.35 AB type	H-20, H-22		129.81[l] d	C-19, C-22
22		2.02 (2H) m	H-21, H-23		27.17[k] t	C-20, C-21, C-23, C-24
23		1.29-1.35[h] m	H-22, H-24		22.32 t	C-21, C-22, C-24
24		1.29-1.35[h] m	H-23, H-25		31.93 t	C-22, C-23
25		0.90 (3H) t (7.1)	H-24		13.98 q	C-23, C-24
4-OH		2.84 br s	H-4		―――	C-3
5-OH		2.36 br s			―――	

a) Signal assignments were based on the results of DEPT, PFG ^{1}H-^{1}H COSY, PFG HMQC, PFG HMBC and difference NOE experiments. b) Numbers in the column indicate the protons that correlated with the proton on the line in the PFG ^{1}H-^{1}H COSY. c) Numbers in the column indicate the protons at which NOE's were detected in the difference NOE experiment under irradiation at the proton on the line. d) Numbers in the column indicate the carbons that showed HMBC correlations with the proton on the line in the PFG HMBC spectrum. e) and g) The W-form long-range couplings were observed between H*e*-2 and H*e*-6 and between H*a*-6 and H*e*-7 respectively in the PFG ^{1}H-^{1}H COSY. f) Negative NOE's were observed on H*e*-2 and H*e*-6 in the difference NOE experiment under irradiation at H-4. h), i) and j) The signals could not be assigned exactly because of the signal overlapping. k) and l) Signal assignments may be interchanged between two signals with the same superscript.
